# Mitigating the Rock‐Salt Phase Transformation in Disordered LNMO Through Synergetic Solid‐State AlF_3_/LiF Modifications

**DOI:** 10.1002/advs.202515962

**Published:** 2025-12-12

**Authors:** Xingqi Chang, Carlos Escudero, Ashley P Black, Sharona Horta, Elías Martínez, Xuan Lu, Jordi Llorca, Maria Ibáñez, Jordi Jacas Biendicho, Andreu Cabot

**Affiliations:** ^1^ Catalonia Institute for Energy Research–IREC Sant Adrià de Besòs Barcelona 08930 Spain; ^2^ Universitat de Barcelona Carrer de Martí i Franquès Barcelona 08028 Spain; ^3^ ALBA Synchrotron Light Source Cerdanyola del Vallès Barcelona 08290 Spain; ^4^ Institute of Science and Technology of Austria (ISTA) Am Campus 1 Klosterneuburg 3400 Austria; ^5^ Department of Chemical Engineering and Center for Research in Multiscale Science and Engineering Universitat Politècnica de Catalunya EEBE Eduard Maristany 10‐14 Barcelona 08019 Spain; ^6^ ICREA Pg. Lluis Companys Barcelona Catalonia 08010 Spain

**Keywords:** disordered spinel LiNi_0.5_Mn_1.5_O_4_ (LNMO), generation 3b batteries, operando SXRD, operando XAS, rock‐salt, solid‐state synthesis

## Abstract

High‐voltage disordered spinel LiNi_0.5_Mn_1.5_O_4_ is a promising cathode material for high power density in lithium‐ion batteries. However, it suffers from poor cycle life associated with the rock‐salt phase transformation. This study presents a straightforward synthesis approach to enhance the electrochemical performance of LiNi_0.5_Mn_1.5_O_4_ through a synergistic solid‐state modification with LiF and AlF_3_. This dual modification promotes rapid Li⁺ diffusion, enables near‐complete delithiation/lithiation, approaching the theoretical capacity of disordered LiNi_0.5_Mn_1.5_O_4_, and, more importantly, effectively mitigates the formation of the rock‐salt phase, thereby enhancing structural stability, as confirmed by *operando* X‐ray absorption spectroscopy (XAS) and synchrotron X‐ray diffraction (SXRD). As a result, the optimized LiNi_0.5_Mn_1.5_O_4_ (10 mg AlF_3_ + 30 mg LiF) delivers high reversible capacities of 142.1, 139.1, 129.2, 121.6, 110.3, 93.5, and 76.1 mAh∙g^−1^ at 0.2C, 0.5C, 1.0C, 2.0C, 3.0C, 4.0C, and 5.0C, respectively. Full cells using graphite as the anode and a high‐loading cathode exhibit excellent cycling performance. They retain 80% of their capacity after 200 cycles at 0.5C within a voltage window of 3.5–4.9 V with cathode loading of 11 mg∙cm^−2^. The findings of this study will significantly advance high‐power LiNi_0.5_Mn_1.5_O_4_ materials, offering improved battery life and thereby enhancing their potential for practical applications.

## Graphical abstract

The transition between the spinel and rock‐salt phases induces irreversible structural changes in disordered LiNi_0.5_Mn_1.5_O_4_, thereby preventing it from fully releasing its electrochemical capacity during charge/discharge cycling.

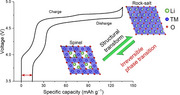

## 1 Introduction

LiNi_0.5_Mn_1.5_O_4_ (LNMO) is a promising cathode material for next‐generation Li‐ion batteries since it operates at a high voltage of ≈4.7 V versus Li/Li^+^, maximizing specific energy density to ≈700 Wh∙kg^−1^ at the cathode material level.^[^ ^1^, ^2^, ^3^ ^]^ As a result, LNMO // graphite full cells can achieve energy densities comparable to those of cells based on LiNi_0.8_Mn_0.1_Co_0.1_O_2_ (NMC811), offering the advantage of the highest power density. This superior power performance is attributed to the 3D Li‐ion diffusion channels inherent to the spinel structure of LNMO, which facilitate fast and efficient delithiation/lithiation during charge–discharge cycles, following the reaction: LiNi^2+^_0.5_Mn^4+^_1.5_O_4_ ⇋ Ni^4+^_0.5_Mn^4+^_1.5_O_4_ + Li^+^ + *e* ^−^.^[^ ^4^, ^5^ ^]^ Additionally, these open channels enable LNMO to undergo nearly complete delithiation/lithiation, thereby maximizing Li resource utilization. This contrasts with commercial cathode materials, such as LiCoO_2_ and NMC811, which utilize only ≈60% and 67% of their theoretical capacities, resulting in Li loss.^[^ ^6^, ^7^, ^8^ ^]^ Therefore, developing LNMO materials is important for effectively utilizing lithium resources.

Two different LNMO crystal structures can form depending on the distribution of Ni and Mn atoms within the lattice.^[^ ^9^ ^]^ These two phases typically coexist in most LNMO cathodes, with their relative abundance strongly influenced by synthesis parameters, particularly the annealing conditions.

The first is the disordered LNMO phase, adopting the non‐stoichiometric AB_2_O_4_ structure within *Fd‐3m* space group, where A‐site Li⁺ occupies the 8*a* sites, B‐site Ni^2+^/Mn^4+^ reside in the 16*d* sites, and O^2−^ are positioned at the 32*e* sites, forming a cubic close‐packed (ccp) oxygen array. The random distribution of Ni and Mn within the B‐sites introduces structural disorder and high symmetry.^[^ ^10^ ^]^ This structure features unoccupied 16*d* octahedral positions, creating zigzag 3D Li‐ion diffusion pathways (8*a*–16*d*–8*a*–16*d*) that enable rapid and complete Li⁺ intercalation/deintercalation during cycling.^[^ ^10^ ^]^ However, disordered LNMO is often non‐stoichiometric and contains Mn^3+^ and oxygen vacancies. These can influence the reaction mechanism and the electrochemical performance of the cathode:^[^ ^11^ ^]^ Mn^3+^ and oxygen vacancies enhance Li⁺ and electronic conductivity, improving rate capability and cycling stability.^[^ ^12^, ^13^, ^14^, ^15^, ^16^ ^]^ It also introduces a major drawback. Mn^3+^ is prone to disproportionation, following the reaction: 2Mn^3+^ → Mn^4+^ + Mn^2+^, where Mn^4+^ remains in the lattice, while Mn^2+^ dissolves into the electrolyte, accelerating capacity fading and cell degradation. Jahn‐Teller distortion further aggravates this issue, which weakens Mn─O bonds, making Mn^3+^ more susceptible to disproportionation.^[^ ^17^, ^18^ ^]^ Additionally, Mn^2+^ can migrate to the anode side, leading to a continuous performance deterioration in graphite‐anode full cells, which limits the practical application of LNMO.^[^ ^19^, ^20^ ^]^ The amount of the disordered phase directly influences the Mn^3+^ content and oxygen vacancy concentration.^[^ ^21^, ^22^, ^23^ ^]^

The second crystal structure is the ordered LNMO phase, which belongs to the *P4_3_32* space group. The Ni^2+^ and Mn^4+^ are distinctly positioned at the 4*a* and 12*d* octahedral sites, respectively. This ordered arrangement prevents the formation of Mn^3+^ and oxygen vacancies, leading to a more stable structure.^[^ ^24^ ^]^ However, Li⁺ diffusion in this phase is slower, and Li^+^ extraction primarily involves the oxidation of Ni^2+^ to Ni^4+^, while Mn^4+^ remains electrochemically inactive.^[^ ^25^ ^]^ The ordered *P4_3_32* LNMO phase offers three key advantages: i) an enhanced output voltage, as it undergoes redox process at 4.75 V corresponding to the Ni^2+^/Ni^4+^ transition, eliminating the 4.1 V plateau associated with the Mn^3+^/Mn^4+^ redox; ii) a higher discharge capacity at low currents, approaching the theoretical limit; and iii) superior resistance to over‐discharge, as the ordered structure undergoes a reversible crystal transformation between full lithiation and delithiation.^[^ ^9^, ^26^, ^27^ ^]^

*Operando* techniques provide direct evidence of a battery's chemical and structural changes during charge/discharge cycling, affording valuable insights into the reaction mechanism. This can be characterized as a function of cation ordering^[^ ^28^ ^]^ or sample stoichiometry using synchrotron and neutron‐based techniques. In disordered Li_1‐_ *_x_* Ni_0.5_Mn_1.5_O_4_, the spinel experiences a solid‐solution reaction within the 0 < *x* <0.5 range, which is associated with the Ni^2+^/Ni^3+^ redox couples. When *x* surpasses 0.5, the disordered phase transforms to rock‐salt Ni_0.25_Mn_0.75_O_2_, which is linked to the Ni^3+^/Ni^4+^ redox couple.^[^ ^29^ ^]^ In contrast, the ordered Li_1‐_ *_x_* Ni_0.5_Mn_1.5_O_4_ spinel endures two‐phase reactions throughout the entire range (0 < *x* < 1), corresponding to the topotactic cubic/cubic phase evolutions between LiNi_0.5_Mn_1.5_O_4_/Li_0.5_Ni_0.5_Mn_1.5_O_4_ and Li_0.5_Ni_0.5_Mn_1.5_O_4_/ Ni_0.5_Mn_1.5_O_4._^[^ ^30^ ^]^ The nucleation and growth of the rock‐salt phase, along with the movement of the grain boundaries, restrict the cathode's kinetics while increasing the risk of the structure's irreversible changes.^[^ ^31^ ^]^

The disordered *Fd‐3m* LNMO phase exhibits superior rate performance compared to the ordered *P4_3_32* phase, primarily due to its faster Li⁺ diffusion.^[^ ^11^, ^27^, ^32^, ^33^, ^34^ ^]^ However, this performance advantage comes at the cost of structural stability, as the disordered phase is more prone to degradation, leading to irreversible capacity loss. In contrast, the ordered LNMO structure offers greater structural integrity and the potential for higher energy density, making it a strong candidate for electric vehicle and portable energy storage applications. Extensive research has focused on combining the advantages of ordered and disordered phases to improve capacity retention and optimize Li^+^ diffusion in LNMO. Various strategies have been explored to achieve this goal, including nanostructure design, structural regulation, coating, and doping, with disordered LNMO as the primary material due to its superior Li^+^ diffusion properties.^[^ ^35^, ^36^ ^]^ These approaches significantly enhance electrochemical performance in terms of rate capability, cycling stability, reduced impedance, and structural integrity.^[^ ^6^ ^]^ However, the inability of disordered LNMO to achieve full delithiation/lithiation limits its suitability for practical applications.^[^ ^27^ ^]^ Besides, these modification techniques have proven ineffective in increasing capacity, as the addition of doping or coating materials reduces the cathode's active mass fraction, thereby diminishing its overall capacity.^[^ ^37^ ^]^

LNMO also suffers from severe electrolyte oxidation and transition‐metal dissolution, resulting in rapid capacity fading.^[^ ^38^ ^]^ Electrolyte oxidation typically produces inorganic (LiF and Li*x*PF*y*O*z*) and organic (polyethers, carbonates) compounds,^[^ ^39^ ^]^ which accumulate on the LNMO surface to form a cathode electrolyte interphase (CEI) layer.^[^ ^40^ ^]^ Thickening of this CEI layer during cycling increases electrode impedance. To mitigate these degradation processes, surface modification has proven to be one of the most effective approaches, as it forms a protective barrier that suppresses side reactions between the LNMO electrode and electrolyte.^[^ ^34^, ^41^ ^]^ Metal fluoride coatings (e.g., AlF_3_, LiF, and MgF_2_) have recently emerged as superior surface modification materials for LNMO due to their exceptionally high chemical stability, strong metal‐fluorine bonding, and lower Gibbs free energy of formation compared with their corresponding oxides.^[^ ^42^ ^]^ These properties effectively suppress electrolyte oxidation, minimize transition metal dissolution, and stabilize the CEI layer during cycling. Gao et al. first reported AlF_3_‐coated LNMO synthesized via a wet chemical route, demonstrating improved electrochemical performance. Wu et al.^[^ ^43^ ^]^ further confirmed that MgF_2_ coating significantly enhanced cycling stability, achieving 89.9% capacity retention after 100 cycles compared with only 69.3% for pristine LNMO. The MgF_2_ layer was shown to inhibit CEI growth and preserve the structural integrity of the spinel, highlighting the ability of fluoride coatings to provide both thermal and electrochemical protection.^[^ ^44^ ^]^

Modification strategies can significantly enhance the electrochemical stability of disordered LNMO, but they simultaneously constrain its fully reversible, full delithiation/lithiation behavior. Whereas ordered LNMO allows nearly complete lithium extraction/insertion and delivers a theoretical capacity of ≈147 mAh∙g^−1^—albeit with poor rate performance—disordered LNMO provides only ≈130 mAh∙g^−1^ (≈88% extraction/insertion) but exhibits superior electrochemical kinetics.^[^ ^45^ ^]^ Moreover, surface modifications reduce the effective capacity by displacing active material. Thus, a central challenge is to integrate the advantages of both ordered and disordered structures to achieve full delithiation together with stable electrochemical performance.

In this work, we present a strategy to tune the structural and physicochemical properties of LNMO through modifications with AlF_3_ and LiF, resulting in enhanced LNMO performance. The dual‐modification approach enables complete and fully reversible delithiation/lithiation, improves Li^+^ diffusion, and ultimately enhances the electrochemical stability of LNMO by modifying the cathode reaction mechanism. To elucidate the reaction mechanism in the optimized LNMO sample, we investigate the valence states and spatial distribution of Ni and Mn simultaneously using *operando* synchrotron X‐ray absorption spectroscopy (XAS) and synchrotron X‐ray diffraction (SXRD). The structural evolution is also examined using high‐resolution transmission electron microscopy (HRTEM), Fourier‐transform infrared (FTIR), and Raman spectroscopy. These comprehensive characterizations reveal the mitigation of the structural transformation in the fully charged state, attributed to the emergence of a rock‐salt phase.

## 2 Results and Discussion

LNMO obtained from TOPSOE Ltd. was combined with small amounts of AlF_3_ and LiF through ball milling (see details in the Experimental Section of the Supporting Information). All samples were produced using 2.0 g of LNMO and various ratios of AlF_3_ and LiF were investigated (Table S1, Supporting Information): LNMO‐1 (40 mg AlF_3_), LNMO‐2 (30 mg AlF_3_ + 10 mg LiF), LNMO‐3 (20 mg AlF_3_ + 20 mg LiF), LNMO‐4 (10 mg AlF_3_ + 30 mg LiF), LNMO‐5 (40 mg LiF), LNMO‐Al (10 mg AlF_3_), and LNMO‐Li (30 mg LiF). The mixtures were ball‐milled at 300 rpm for 4 h, followed by annealing in air at 800 °C for 4 h (Figure S1, Supporting Information). A bare LNMO sample annealed in air at 800 °C for 4 h was also characterized for comparison purposes (LNMO‐Air in Table S1, Supporting Information).

**Figure 1:**
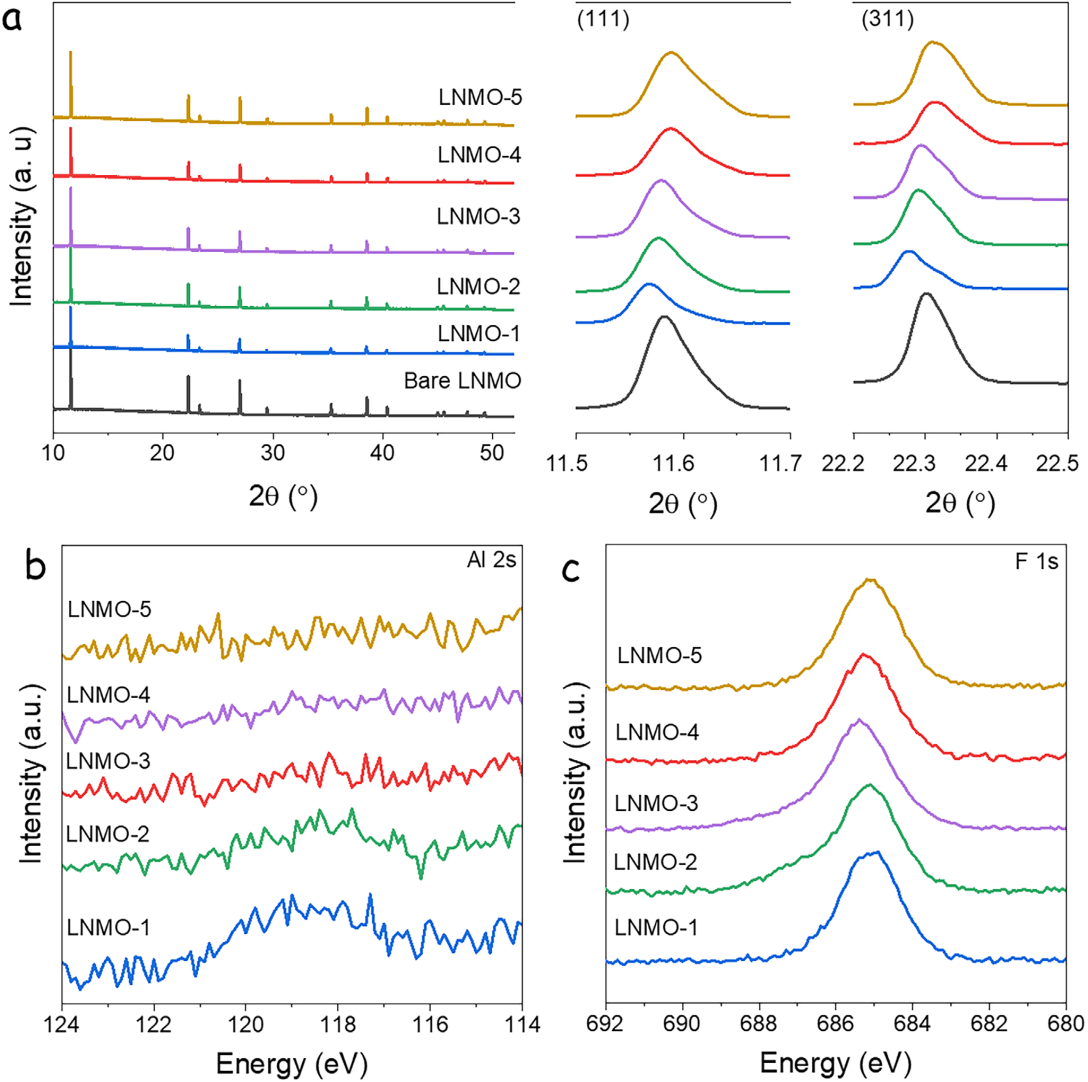
a) SXRD patterns and (111) and (311) peak magnification, b) XPS spectra of Al 2s, and c) F 1s for the modified and bare LNMO samples.

**Figure** **1**a presents the SXRD patterns of LNMO samples collected at the NOTOS beamline at ALBA synchrotron. Patterns exhibit main diffraction peaks at 2*θ* = 11.6°, 22.2°, 27.0°, and 38.5° (*λ* = 0.954 Å), corresponding to the (111), (311), (400), and (511) planes of the cubic LNMO phase. No extra peaks corresponding to AlF_3_ or LiF are observed in the diffraction patterns, indicating that the salts fully reacted with LNMO. Compared to bare LNMO, the SXRD peaks in LNMO‐1 and LNMO‐2 (samples synthesized mainly with AlF_3_) shift slightly toward lower angles. In contrast, those in LNMO‐4 and LNMO‐5 (samples synthesized mainly with LiF) shift to the right, indicating lattice parameter variations after reaction with AlF_3_ and LiF. Further analysis of the phase evolution in LNMOs was conducted by Rietveld refinement of the crystal structure using SXRD data (Figure S2, Supporting Information). Two structural models were considered: a single‐phase refinement using the F3‐dm phase, and a two‐phase refinement considering both the F3‐dm and P43_3_2 phases. The resulting lattice parameters are shown in Table S2 (Supporting Information). The single‐phase model provides a better fit (Rp and χ^2^) to all data sets. The refined lattice parameters are 8.1782, 8.1779, 8.1736, 8.1697, and 8.1657 Å for LNMO‐1, LNMO‐2, LNMO‐3, LNMO‐4, and LNMO‐5, while the parameter of bare LNMO is 8.1725 Å. The AlF_3_ and LiF modifications change the crystal structure of bare LNMO; the former promotes lattice expansion, whereas the latter contracts the LNMO structure. X‐ray photoelectron spectroscopy (XPS) analysis was conducted to investigate the chemical composition at the surface level. The measured spectra, spanning the binding energy range from 124 to 114 eV and 692 to 680 eV, are shown in Figure 1b,c, respectively. The former range corresponds to the Al 2*s* region and the latter to the F 1*s*. The Al signal decreases systematically from LNMO‐1 to LNMO‐5, while the F 1*s* region displays a strong contribution centered ≈685 eV.

**Figure 2:**
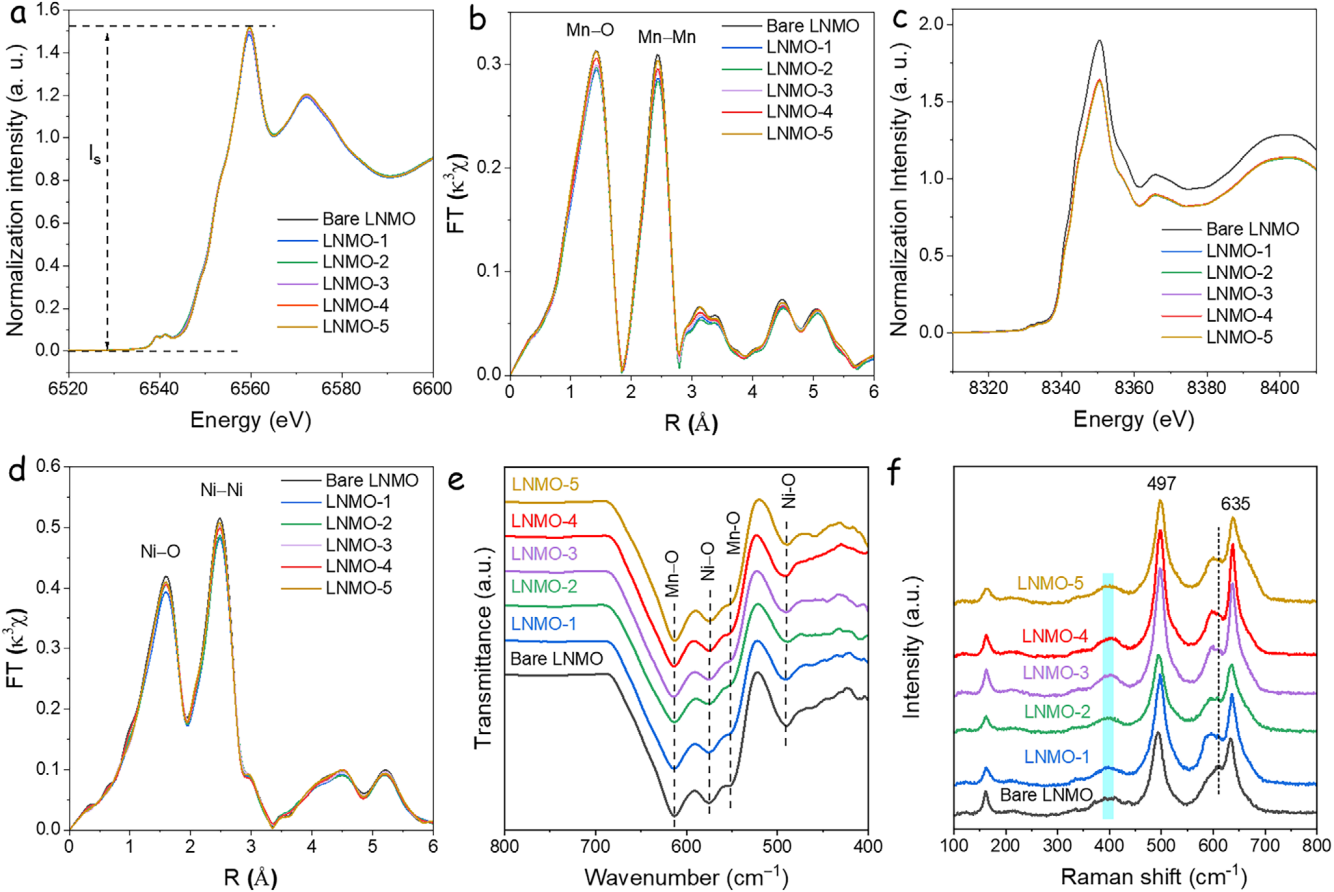
a) Mn K‐edge XANES spectra and b) FT‐EXAFS (κ3χ) in R space for Mn K‐edge. c) Ni K‐edge XANES spectra and d) FT‐EXAFS (κ3χ) in R space for Ni K‐edge. e) FTIR spectra. f) Raman spectra.

To investigate the local structure of transition metal atoms, the Mn and Ni K‐edge X‐ray absorption near‐edge structure (XANES) and Fourier‐transformed (FT) extended X‐ray absorption fine structure (FT‐EXAFS) spectra were analyzed (**Figure** **2**a–d). For the Mn K‐edge, Figure 2a,b, the modified LNMO samples display a similar edge profile. The pre‐edge peaks are attributed to the forbidden 1*s* to 3*d* transition, and peak intensity variation indicates changes in the metal site proportion in [MnO_6_] octahedral.^[^ ^46^, ^47^ ^]^ As shown in Figure S3 (Supporting Information), two peaks in the pre‐edge are observed, and the LNMO‐1 (40 mg AlF_3_) exhibits the highest intensity, while the bare LNMO displays a weaker peak intensity.^[^ ^48^ ^]^ The FT‐EXAFS spectra (Figure 2b) reveal that the primary peaks corresponding to the Mn─O and Mn─Mn bonds are consistently located at 1.4 and 2.4 Å (Figure S4, Supporting Information), respectively, in agreement with previous XAS studies.^[^ ^28^ ^]^ The intensity of the Mn K‐edge FT‐EXAFS contribution (κ3χ, Figure S5, Supporting Information), which is related to the local structure around the Mn atoms in the [MnO_6_] octahedral structure,^[^ ^49^, ^50^ ^]^ is affected by the AlF_3_ and LiF modifications.

The XANES and pre‐edge features of the Ni K‐edge are shown in Figure 2c and Figure S6 (Supporting Information), respectively, with bare LNMO showing the lowest oxidation state among the series. The LNMO samples modified with AlF_3_ and LiF exhibit a higher oxidation state for Ni. The FT‐EXAFS spectra in R‐space (Figure 2d; Figure S7, Supporting Information) reveal two prominent peaks corresponding to Ni^2+^─O and Ni─Ni bonds.^[^ ^28^ ^]^ Additional insights from Figure S8 (Supporting Information) show a decrease in the intensity of both Ni─bond peaks in FT‐EXAFS spectra, from bare LNMO to LNMO‐1.

IR spectroscopy is an effective tool for distinguishing between the *P4_3_32* and *Fd‐3m* phases of LNMO. According to Amatucci et al.,^[^ ^23^ ^]^ these two spinel structures can be differentiated by: 1) the number of IR absorption bands, eight for the ordered *P4_3_32* phase and five for the disordered *Fd‐3m* phase, and 2) the relative intensity ratios among the absorption peaks.^[^ ^51^ ^]^ Figure 2e shows four distinct FTIR absorption peaks at 613, 574, 551, and 490 cm^−1^. The bands at approximately 613 and 574 cm^−1^ correspond to Mn─O and Ni─O stretching vibrations within the [MnO_6_] octahedral framework, respectively. Notably, the 613 cm^−1^ band exhibits greater intensity than the 574 cm^−1^ band, consistent with the disordered *Fd‐3m* phase assignment.^[^ ^34^ ^]^ The peaks at 551, 458, and 430 cm^−1^ are characteristic of the ordered *P4_3_32* phase.^[^ ^6^, ^34^ ^]^ Additionally, cation disordering in LNMO can be semi‐quantitatively assessed by analyzing the intensity ratio of the 613 to 574 cm^‒1^ peaks (*I_613_*/*I_574_*), which also captures Mn/Ni ordering effects.^[^ ^52^, ^53^, ^54^, ^55^ ^]^ The calculated *I_613_*/*I_574_* ratios for the series are 1.10 (bare LNMO), 1.18 (LNMO‐1), 1.14 (LNMO‐2), 1.15 (LNMO‐3), 1.14 (LNMO‐4), and 1.13 (LNMO‐5), indicating small variations in cation ordering for the modified samples compared to bare LNMO. The Raman spectra (Figure 2f) trends are consistent with the infrared data. The prominent peak ≈635 cm^−1^ corresponds to the symmetric Mn─O stretching vibration (A_1g_ mode) of the [MnO_6_] octahedra, while the bands near 400 and 494 cm^−1^ are attributed to Ni^2+^─O stretching modes within the spinel structure.^[^ ^51^ ^]^ A peak in the 580–606 cm^‒1^ region is associated with the T_2g_ mode of the ordered *P4_3_32* phase; this peak exhibits a leftward shift and increased intensity compared to the bare LNMO, suggesting that the modified LNMO samples exhibit a higher degree of ordered structure. Additional characteristic peaks of the ordered *P4_3_32* symmetry typically appear in the 200–250 cm^‒1^ range. Still, these are absent across all samples, confirming that the dominant phase in these LNMO materials is the disordered *Fd‐3m* phase.^[^ ^6^, ^56^ ^]^

**Figure 3:**
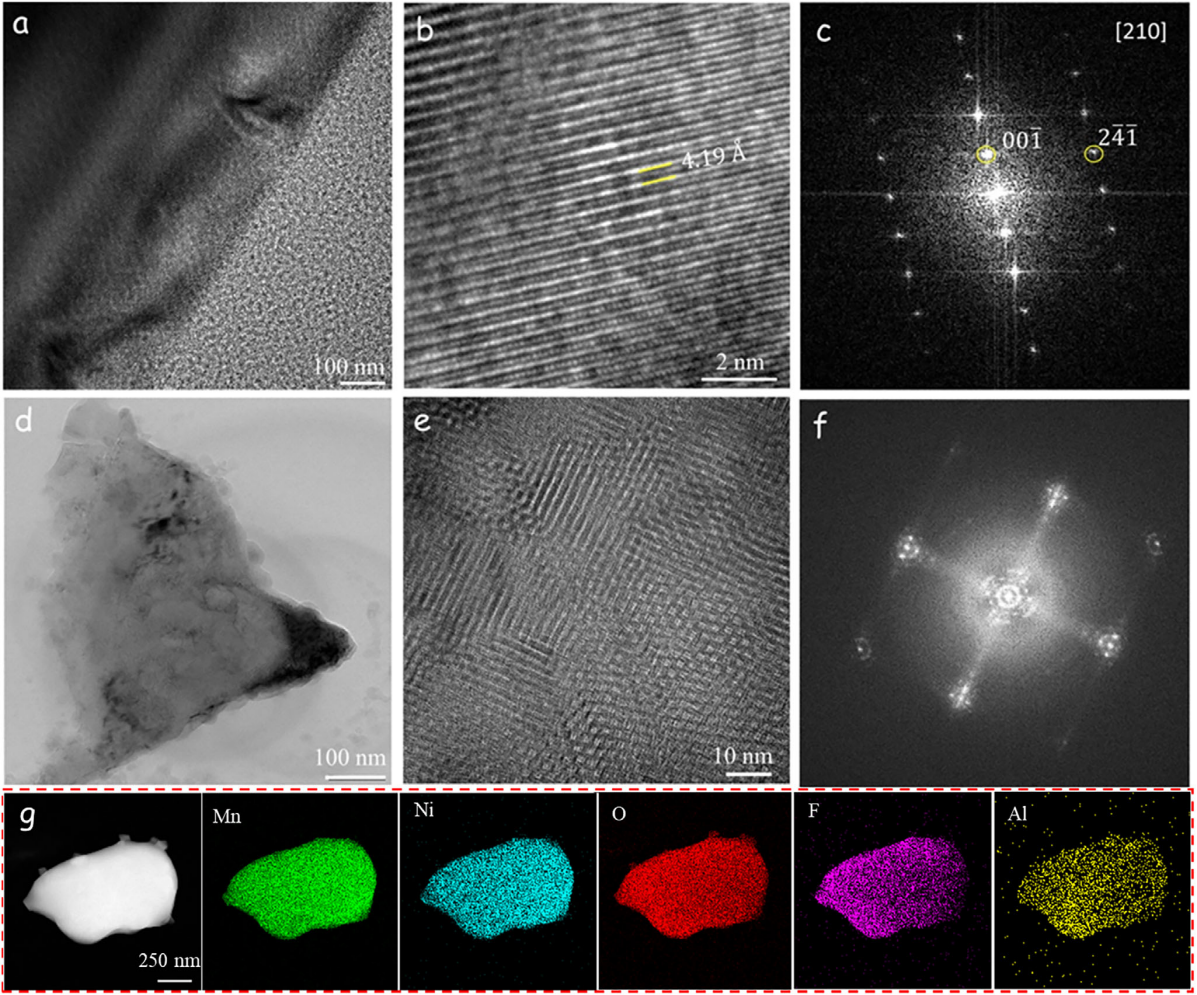
TEM images of LNMO‐4. a) TEM image of the surface of an LNMO particle. b) HRTEM image and c) SAED pattern performed along the [210] zone axis, revealing a truncated octahedral spinel structure. d) TEM image of LNMO particle. e) HRTEM image of short‐range atomic ordering, f) corresponding to the SAED patterns; g) EDS mappings.

**Figure** **3**a–g presents representative transmission electron microscopy (TEM) and HRTEM images, and selected area electron diffraction (SAED) patterns of the highly crystalline LNMO‐4. For comparison, the TEM analysis of bare LNMO is provided in Figure S9 (Supporting Information). The observed lattice fringes reveal an interplanar spacing of 4.19 Å for the (210) plane in LNMO‐4, while bare LNMO exhibits a spacing of 4.7 Å for the (111) plane. The SAED patterns confirm the spinel structure (*Fd‐3m* space group), in which Ni and Mn are randomly disordered within the 16*d* octahedral sites.^[^ ^34^ ^]^ Notably, LNMO‐4 exhibits regions with short‐range atomic ordering (Figure 3e,f), a feature absent in bare LNMO. In localized areas, the SAED patterns of LNMO‐4 display additional diffraction peaks associated with the *P4_3_32* phase, suggesting an increase in the ordered phase,^[^ ^57^ ^]^ due to AlF_3_ and LiF modifications. Furthermore, energy‐dispersive spectroscopy (EDS) elemental mapping (Figure 3g) reveals the distribution of O, F, Al, Mn, and Ni, indicating the homogeneous introduction of fluorine and aluminum in LNMO‐4. The morphology and size of the secondary particles are preserved after modification using AlF_3_ and LiF.

In summary, the solid‐state reaction of LNMO with different amounts of AlF_3_ and LiF has relevant effects. The reaction modifies both the crystal structure and surface chemistry of LNMO. The XANES and diffraction spectra suggest that AlF_3_ and LiF modulate the chemical state of Ni, and AlF_3_ promotes lattice expansion, whereas LiF contracts the LNMO structure. At the surface level, XPS and EDS analyses detect Al and F on the primary particles, which preserve the truncated polyhedral shape from bare LNMO. Additionally, IR and Raman spectroscopy techniques support an averaged disordered *Fd‐3m* structural model for the modified samples with ordered domains for the LNMO‐4 sample, as evidenced by TEM analysis. These modifications collectively influence the local symmetry, electronic structure, and electrochemical behavior of the LNMO materials.

**Figure 4:**
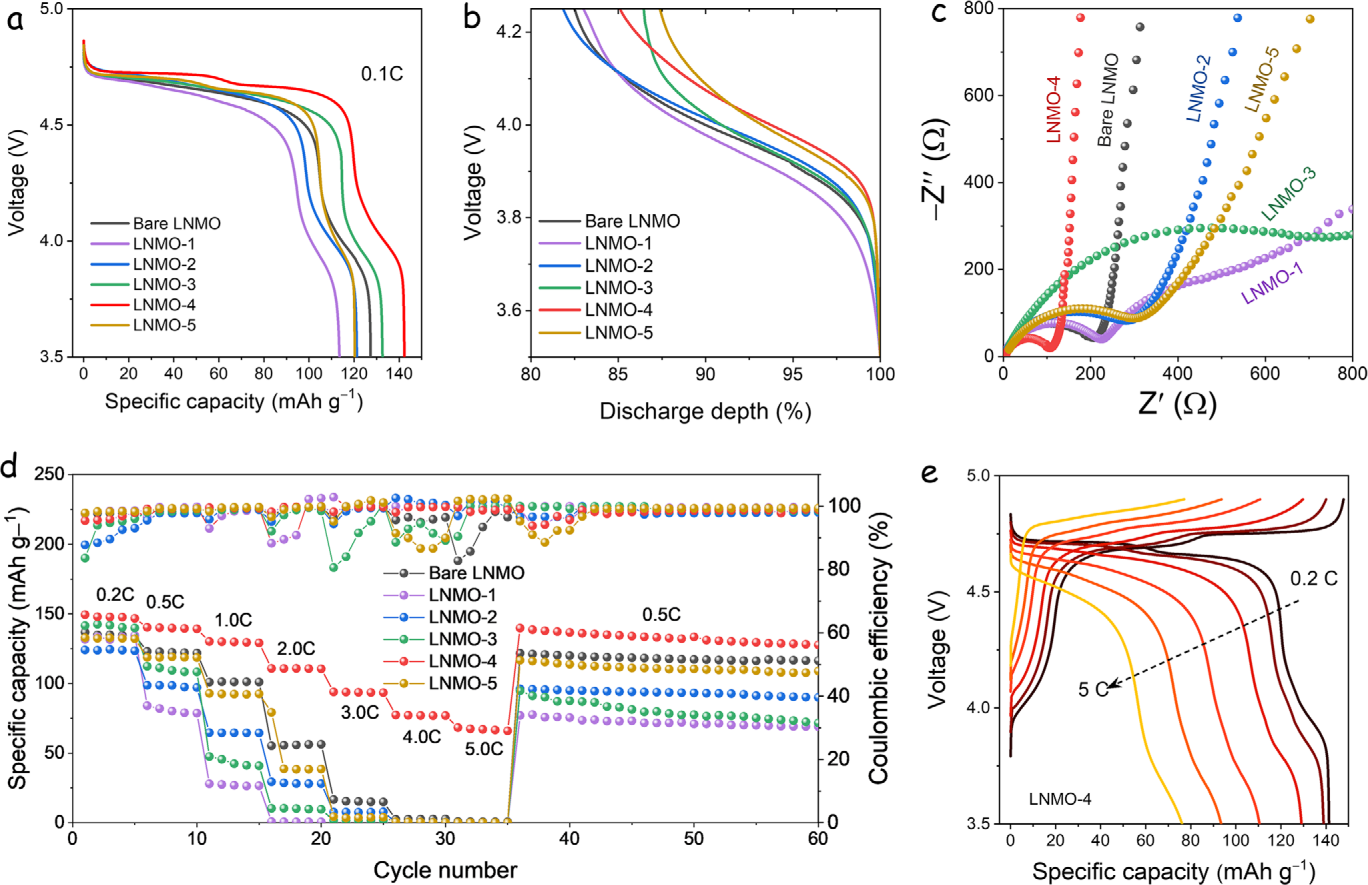
Electrochemical performance of bare LNMO, LNMO‐1, LNMO‐2, LNMO‐3, LNMO‐4, and LNMO‐5 electrodes. a) The initial discharge profiles. b) The normalized initial capacity profiles are between 3.5 and 4.2 V. c) Nyquist plots of EIS after 50 cycles carried out at a 3.5 V discharge potential. d) Rate performance. e) GCD profiles of LNMO‐4 at different current densities.

The electrochemical performance of the LNMO samples was evaluated at room temperature using CR2032 coin‐type cells, with a minimum cathode mass loading of 4.0 mg∙cm^‒2^. The initial discharge profiles at a current rate of 0.1C are presented in **Figure** **4**a (1C = 147 mA∙g^‒1^). All samples display the characteristic electrochemical behavior of disordered spinel.^[^ ^26^ ^]^ Among them, the LNMO‐4 electrode exhibits the highest initial discharge capacity of 142 mAh∙g^‒1^, outperforming the bare LNMO (128 mAh∙g^‒1^), LNMO‐1 (113 mAh∙g^‒1^), LNMO‐2 (122 mAh∙g^‒1^), LNMO‐3 (132 mAh∙g^‒1^), and LNMO‐5 (120 mAh∙g^‒1^). The redox reactions occurring within the LNMO system offer insights into the Mn^3+^ content, which can be semi‐quantitatively evaluated by analyzing the capacity contribution of the Mn^4+^/Mn^3+^ redox couple near the ≈4.0 V plateau.^[^ ^57^ ^]^ We quantified this contribution in the 3.5–4.2 V voltage range during the initial discharge at 0.1C (Figure 4b). The resulting Mn^4+^/Mn^3+^ redox contributions to the total discharge capacity are 17.0% (bare LNMO), 17.5% (LNMO‐1), 18.2% (LNMO‐2), 13.5% (LNMO‐3), 14.9% (LNMO‐4), and 12.6% (LNMO‐5). Interestingly, LNMO‐5, which contains only LiF, exhibits the lowest Mn^3+^ content, consistent with the pre‐edge features and lattice parameter trends observed in the XAS and SXRD analyses.

Figure 4c shows the Nyquist plots obtained from electrochemical impedance spectroscopy (EIS) for the different LNMO cells, measured at the fully discharged state (3.5 V vs Li⁺/Li) after 50 cycles. Each spectrum displays a depressed semicircle in the medium‐frequency region, corresponding to the charge transfer resistance at the electrode–electrolyte interface. This is followed by a sloped line in the low‐frequency region, which reflects the Warburg impedance associated with Li^+^ diffusion within the bulk electrode.^[^ ^58^ ^]^ An equivalent circuit for fitting the impedance data is shown in Figure S11 (Supporting Information). The impedance data reveal that the LNMO‐4 sample exhibits a lower charge transfer resistance, indicating a more stable and conductive cathode‐electrolyte interphase (CEI).^[^ ^59^ ^]^The calculated values using equivalent circuit analysis were 55.68, 84.34, 39.43, 229.91, 20.63, and 112.4 Ω, for bare LNMO, LNMO‐1, LNMO‐2, LNMO‐3, LNMO‐4, and LNMO‐5, respectively. In addition, the steeper Warburg slope suggests a higher Li‐ion diffusion coefficient, which is beneficial for efficient Li‐ion transport and contributes to the superior electrochemical performance of LNMO‐4.

Figure 4d presents the rate capability of the LNMO samples, evaluated by discharging at rates ranging from 0.2C to 5.0C, while maintaining a constant charging rate of 0.5C. The LNMO‐4 electrode performs best, delivering capacities of 142.1, 139.1, 129.2, 121.6, 110.3, 93.5, and 76.1 mAh∙g^‒1^ at 0.2C, 0.5C, 1.0C, 2.0C, 3.0C, 4.0C, and 5.0C, respectively. The corresponding galvanostatic charge–discharge (GCD) profiles (Figure 4e) demonstrate that LNMO‐4 retains the highest specific capacities across all C‐rates, highlighting its excellent rate performance. After rate tests, LNMO‐4 maintains a discharge capacity of 132.8 mAh∙g^‒1^ at 0.5C (Figure 4d). In comparison, the discharge capacities of the bare LNMO, LNMO‐1, LNMO‐2, LNMO‐3, and LNMO‐5 electrodes are 118.5, 96.9, 93.3, 119.3, and 103.4 mAh∙g^‒1^, respectively. The high capacity of LNMO‐4 approaches the theoretical limit, indicating that lithium ions can nearly fully intercalate within its reversible structure during discharge.

Galvanostatic intermittent titration technique (GITT) measurements for the bare LNMO and LNMO‐4 electrodes were carried out with a 5 min relaxation period during each galvanostatic step at 0.5C, as shown in Figure S12 (Supporting Information).^[^ ^60^ ^]^ The LNMO‐4 electrode demonstrates enhanced Li^+^ diffusion kinetics, with diffusion coefficients ranging from 10^−9^ to 10^−8^ cm^2^·s^−1^, while the bare LNMO shows significantly lower diffusivity, in the range of 10^−11^–10^−8^ cm^2^·s^−1^ (Figure S8b, Supporting Information). Moreover, the lower relaxation potentials observed for LNMO‐4 during both charge and discharge steps suggest reduced polarization, which is expected to contribute to a higher average operating voltage and improved energy density. These findings further support the superior electrochemical performance of LNMO‐4, characterized by a high specific capacity and fast Li^+^ diffusion.

To isolate and better understand the individual contributions of AlF_3_ and LiF to the enhanced performance of the LNMO‐4 sample, three additional reference electrodes were prepared using the same synthesis method, but with selective fluorine sources: no fluorine (LNMO‐Air), 10 mg of AlF_3_ (LNMO‐Al), or 30 mg of LiF (LNMO‐Li). As expected, XRD shows LNMO‐Al's (111) peak shifting to lower angles compared to bare LNMO and LNMO‐Air, while LNMO‐Li's peak shifts slightly to higher angles (Figure S13, Supporting Information), consistent with SXRD results presented in Figure 1a. The Raman spectrum also confirms a disordered crystal structure for LNMO‐Li, LNMO‐Al, and LNMO‐Air (Figure S14, Supporting Information).

**Figure 5:**
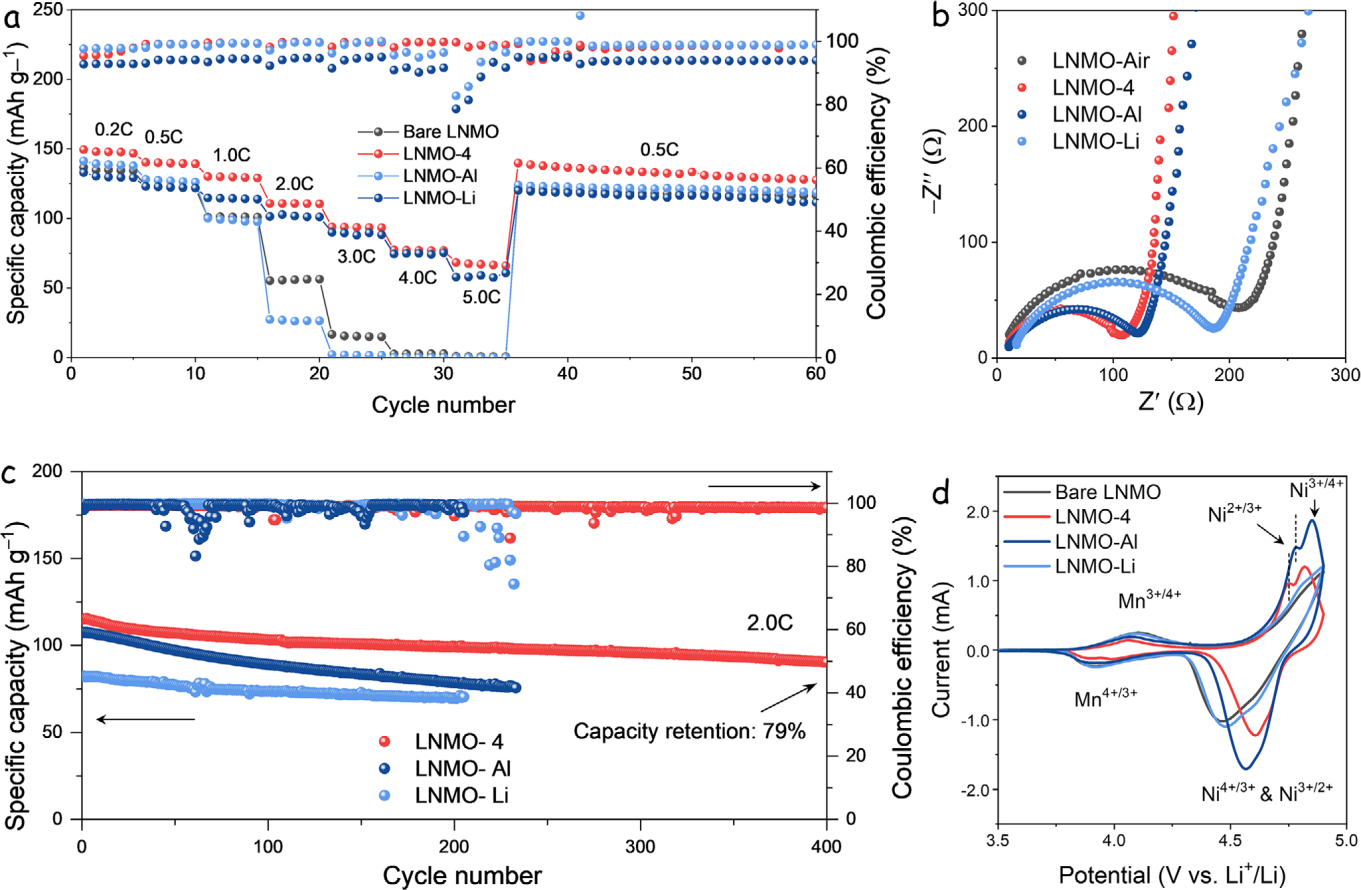
Electrochemical performance of LNMO‐Air, LNMO‐4, LNMO‐Al, and LNMO‐Li cathodes. a) Rate cycling performance at 0.5C and b) the Nyquist plot of EIS tests after 50 cycles at 0.5C, carried out at a 3.5 V discharge potential. c) Cycling performance at 2.0C. d) CV curves at a scan rate of 0.2 mV s^−1^.

**Figure** **5**a shows the cycling performance of these samples at different C‐rates. LNMO‐Al and LNMO‐Li exhibit higher discharge capacities than LNMO‐Air, indicating that each fluoride additive independently improves electrochemical stability. Additionally, comparing the rate performance between LNMO‐Li and LNMO‐Al reveals similar discharge capacities at 0.5C, but a significantly enhanced performance for LNMO‐Al at higher C‐rates (≥2.0C). On the other hand, LNMO‐Air shows inferior capacity retention at higher C‐rates, indicating that the re‐annealing process alone does not contribute positively to the electrochemical performance of LNMO. Figure 5b presents the EIS spectra collected at 3.5 V versus Li⁺/Li after 50 cycles. The LNMO‐4 electrode demonstrates the lowest charge transfer resistance among all samples, indicating that the combined presence of AlF_3_ and LiF produces a synergistic effect that enhances interfacial charge transport and C‐rate performance.

Figure 5c presents the long‐term cycling performance of the LNMO‐4, LNMO‐Al, and LNMO‐Li electrodes at a current rate of 2.0C. The electrodes deliver reversible discharge capacities of approximately 117, 108, and 82 mAh·g^−1^, with capacity retention of 87.3%, 73.8%, and 98.3% after 200 cycles. This demonstrates the sole effect of LiF on the electrochemical stability of LNMO. LNMO‐4 delivers the highest capacity and stable cycling behavior, maintaining 79% of its initial capacity after 400 cycles.

Cyclic voltammetry (CV) was performed on all samples to investigate their redox behavior and electrochemical performance. As shown in Figure 5d, the CV curves (recorded at 0.2 mV·s^−1^ after 50 cycles at 0.5C) exhibit a peak near 4.0 V, corresponding to the Mn^3+^/Mn^4+^ redox couple, and another at ≈4.7 V, assigned to Ni^2+^/Ni^4+^.^[^ ^37^ ^]^ Notably, the anodic peaks of LNMO‐4 and LNMO‐Al split into two distinct features ≈4.7 V, reflecting the highly reversible Ni^2+^/Ni^3+^ and Ni^3+^/Ni^4+^ redox processes.^[^ ^61^ ^]^ This splitting, caused by reduced polarization, suggests improved reaction kinetics in these samples. Further supporting this observation, CV scans at varying rates (Figure S15, Supporting Information) reveal that only LNMO‐Al and LNMO‐4 display well‐defined Ni^3+^/Ni^4+^ redox peaks, further confirming their lower electrochemical polarization compared to other samples.

As reported in previous studies, the LNMO‐Al sample exhibits superior capacity retention across a wide range of C‐rates.^[^ ^62^ ^]^ This can be discussed in terms of 1) an increased Mn^3+^ content in the spinel structure, 2) changes in the lattice parameter *a*, and 3) the chemical modifications at the surface particle level.^[^ ^44^, ^63^ ^]^ In contrast, LNMO‐Li displays a lower rate capability but delivers the highest cycling stability. These results collectively suggest that the LNMO‐4 sample successfully combines the beneficial features of both LNMO‐Li and LNMO‐Al. The synergistic interaction between LiF and AlF_3_ enhances the C‐rate performance and improves electrochemical stability across different operating conditions.

**Figure 6:**
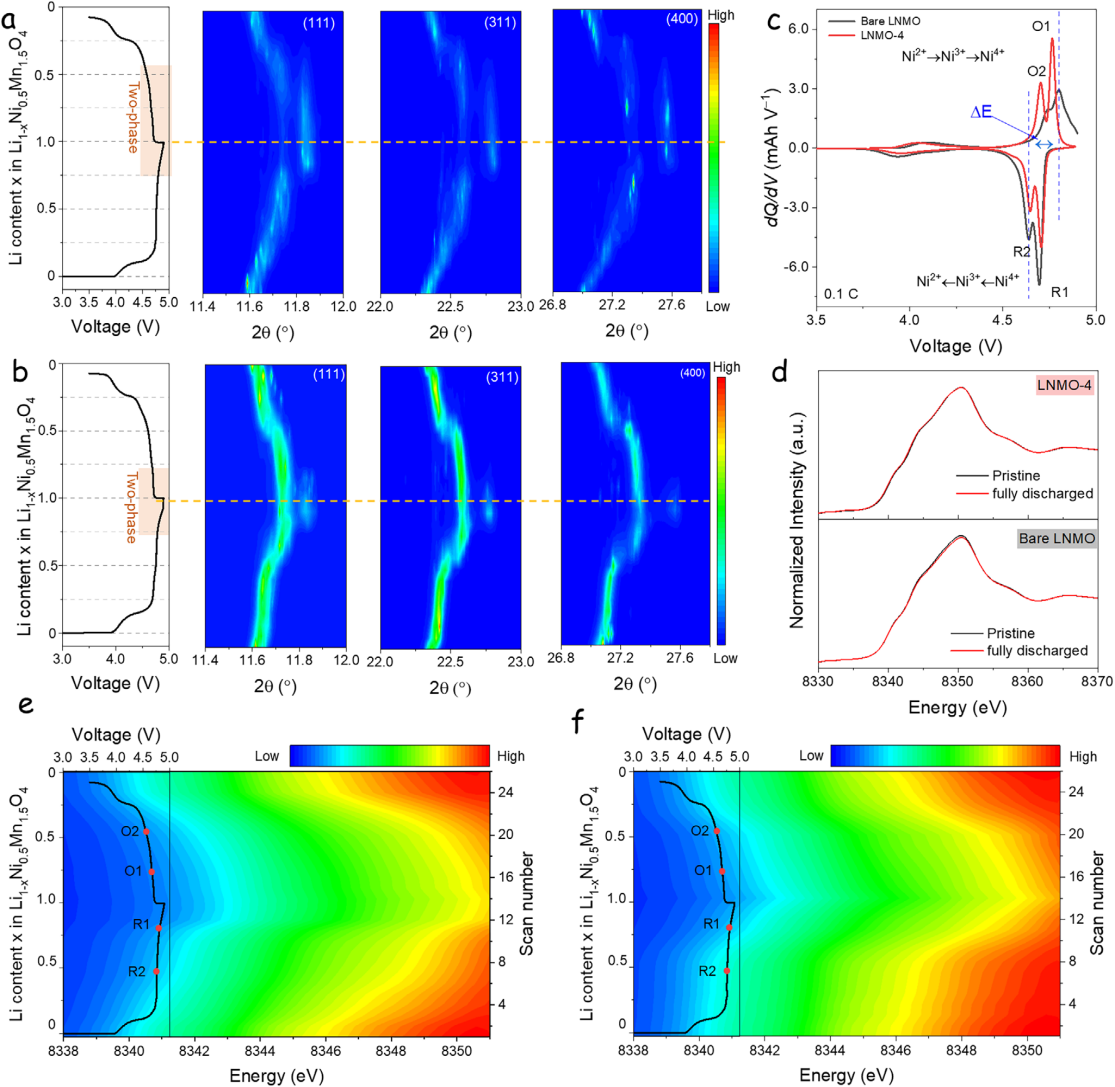
*Operando* SXRD patterns of a) bare LNMO and b) LNMO‐4 electrodes. c) *dQ*/*dV* plots at 0.1C generated from the GCD curves obtained in the *operando* tests. d) Ni K‐edge XANES spectra of fully discharged pristine states of bare LNMO and LNMO‐4 electrodes. e,f) *Operando* Ni K‐edge XANES spectra contour plots based on pre‐edge (green) elaborated using 26 data points for (e) bare LNMO and (f) LNMO‐4.

To elucidate the reaction mechanism of LNMO‐4, *operando* SXRD and XAS (a picture of the experimental setup is shown in Figure S16, Supporting Information) were conducted to monitor structural evolution and transition metal valence state changes during cycling. **Figures** **6**a,b, and S17 (Supporting Information) present the *operando* SXRD results obtained using half‐cells. During the o*perando* tests at a current rate of 0.1C, the bare LNMO and LNMO‐4 electrodes delivered initial discharge capacities of 124 and 131 mAh·g^−1^, respectively. In the *operando* SXRD patterns of both samples, prominent reflections at 2*θ* ≈ 11.6°, 22.5°, and 27.2° correspond to the (111), (311), and (400) crystal planes of the spinel LNMO phase, respectively.

For the bare LNMO electrode (Figure 6a), the (111) peak gradually shifts toward higher angles and diminishes at 4.78 V, indicating lithium extraction and the associated contraction of the spinel lattice. At voltages beyond 4.72 V, new diffraction peaks emerge at 2*θ* ≈ 11.87°, 22.8°, and 27.6°, characteristic of a rock‐salt Ni_0.25_Mn_0.75_O_2_ phase.^[^ ^64^ ^]^ This implies the coexistence of spinel and rock‐salt phases (two‐phase region) during high‐voltage operation.^[^ ^65^, ^66^ ^]^ The rock‐salt peaks appear in the diffraction patterns at Li_0.25_Ni_0.5_Mn_1.5_O_4_ during delithiation and vanish at Li_0.67_Ni_0.5_Mn_1.5_O_4_ during lithiation, so the two‐phase domain covers the high voltage plateau associated with the Ni^4+^→Ni^2+^ redox couple. The formation of this rock‐salt Ni_0.25_Mn_0.75_O_2_ phase is likely due to the migration of transition metal ions into adjacent vacant octahedral sites and the disruption of cation ordering within the spinel lattice.^[^ ^10^, ^67^ ^]^ The rock‐salt phase is stable for a large solubility range (up to Li_0.67_Ni_0.5_Mn_1.5_O_4_ during lithiation)_,_ which compromises the structural stability of the disordered LNMO.

In contrast, upon delithiation of LNMO‐4, Figure 6b, the (111) peak gradually shifts toward higher angles and diminishes near 4.73 V, where the characteristic peaks of the rock‐salt Ni_0.25_Mn_0.75_O_2_ phase appear. During the subsequent discharge process (lithiation), the rock‐salt peak intensity decreases and eventually vanishes ≈4.56 V, corresponding to Li_0.26_Ni_0.5_Mn_1.5_O_4_. The (111) spinel peak spans the entire discharge process, shifting rapidly toward lower angles, indicating Li^+^ reinsertion into the spinel structure.^[^ ^68^ ^]^ Similar reversible shifts and intensity changes are observed for the (311) and (400) reflections, confirming the sample's structural reversibility and phase evolution during cycling.

To define the rock‐salt content in the fully charged states of LNMOs, Rietveld refinement of the bare LNMO and LNMO‐4 crystal structures was conducted using SXRD patterns (Figure S18, Supporting Information). Additionally, the lattice parameters obtained from Rietveld refinement of operando SXRD data are shown in Figure S19 (Supporting Information). At the fully charged state, the LNMO‐4 sample only contains 17% of the rock‐salt phase, whereas the bare LNMO exhibits a much higher fraction of 35%. The lattice parameter evolution of the spinel phase during charging is similar for the two samples and indicates a solid‐solution reaction with a reducing parameter. During discharge, parameter *a* does not behave exactly for the two samples.

The LNMO‐4′s improved stability is attributed to the synergistic effect of AlF_3_/LiF modification, which helps narrow the two‐phase domain and the weight fraction of the rock‐salt phase while maintaining the reversible spinel framework during extended cycling.

Consistent with the *operando* SXRD results, the electrochemical behavior during cycling reflects a similar structural evolution. Figure 6c presents the *dQ/dV* versus voltage plots obtained from GCD profiles during *operando* tests, clearly revealing six characteristic redox peaks that are representative of the disordered spinel structure of LNMO.^[^ ^69^ ^]^ Consistent with the CV data, the anodic and cathodic peaks near 4.1 V correspond to the Mn^3+^/Mn^4+^ redox couple, while redox processes involving Ni^2+^ ↔ Ni^4+^ occur at higher potentials (above 4.5 V). These Ni redox processes can be further resolved into two sequential steps: Ni^2+^ ↔ Ni^3+^ and Ni^3+^ ↔ Ni^4+^, associated with the coexistence of the spinel and rock‐salt phases, and denoted as O2/R2 and O1/R1 peaks, respectively, due to corresponding changes in the spinel lattice parameters. The LNMO‐4 electrode exhibits almost identical oxidation and reduction peak intensities and positions, indicating good reversibility. In contrast, the bare LNMO shows increased intensity in the R1 and R2 peaks compared to O1 and O2. The potential separation (*ΔE*) between anodic and cathodic Ni peaks indicates polarization in the Ni^3+^ ↔ Ni^2+^ redox couple.^[^ ^70^ ^]^ Therefore, LNMO‐4 exhibits a smaller *ΔE*, signifying lower polarization and improved reaction kinetics. This observation aligns with the results from GITT and EIS measurements, which confirm that LNMO‐4 exhibits faster Li+ diffusion and a lower relaxation voltage, consistent with the dominance of the spinel phase throughout the reaction process and a minimized two‐phase domain.

Additional CV profiles recorded for bare LNMO and LNMO‐4 at a scan rate of 0.2 mV·s^−1^ (Figure S20, Supporting Information) show that the bare LNMO electrode exhibits a poorly defined anodic peak at 4.9 V, indicative of an incomplete redox process. This suggests a transition from the spinel to rock‐salt phase through the Ni^2+^/Ni^4+^ redox couple, fast‐passing the intermediate Ni^2+^/Ni^3+^ state, which reflects polarization due to structural instability and two‐phase evolution.^[^ ^71^ ^]^ This behavior highlights the instability of Ni^3+^‐based configurations and contributes to higher electrochemical impedance. Moreover, comparing cathodic peak potentials reveals a clear difference: 4.60 V for LNMO‐4 and 4.46 V for bare LNMO, reflecting greater electrochemical reversibility in LNMO‐4 and higher polarization losses in the bare LNMO electrode.

The Ni K‐edge XANES spectra for both electrodes in their pristine and fully discharged states are shown in Figure 6d. A slight difference is observed in the spectra of the bare LNMO electrode, indicating structural and electronic changes during cycling. In contrast, the LNMO‐4 electrode exhibits nearly identical spectra before and after discharge, suggesting a highly reversible redox process and structural stability during lithiation and delithiation, characteristic of a more robust spinel framework. Figure 6e,f presents the *operando* Ni K‐edge XANES spectra for the bare LNMO and LNMO‐4 electrodes, respectively, focusing on the pre‐edge region (green color). The red markers highlight the Ni redox states corresponding to the *dQ*/*dV* peaks (Figure 6c). Additional *operando* XANES data are provided in Figure S21 (Supporting Information). For the bare LNMO, the Ni K‐edge spectra reveal a discontinuous shift around the Ni^4+^ ↔ Ni^3+^ transition (regions R1 and O1), which correlates with the formation of the rock‐salt phase. This phase transformation is associated with significant changes in Ni atoms' local structure and electronic environment, indicating a spinel framework breakdown.^[^ ^72^ ^]^ In contrast, the LNMO‐4 electrode displays smooth and continuous spectral changes throughout the charge–discharge cycle, suggesting that the spinel structure is better preserved with minimal involvement of irreversible phase transitions, consistent with the *operando* SXRD findings.

In summary, the rock‐salt phase in bare LNMO is stable for an extensive solubility range, up to Li_0.67_Ni_0.5_Mn_1.5_O_4_ during lithiation_,_ compromising the disordered LNMO's structural stability. This increases electrochemical polarization and introduces the risk of irreversible structural degradation, leading to the loss of active Li^+^ storage sites. The AlF_3_/LiF‐modified LNMO‐4 electrode demonstrates a narrower two‐phase domain, up to Li_0.26_Ni_0.5_Mn_1.5_O_4_ during lithiation, with a robust spinel structure along the charge/discharge processes, corresponding to an improved structural reversibility and an enhanced electrochemical performance.

**Figure 7:**
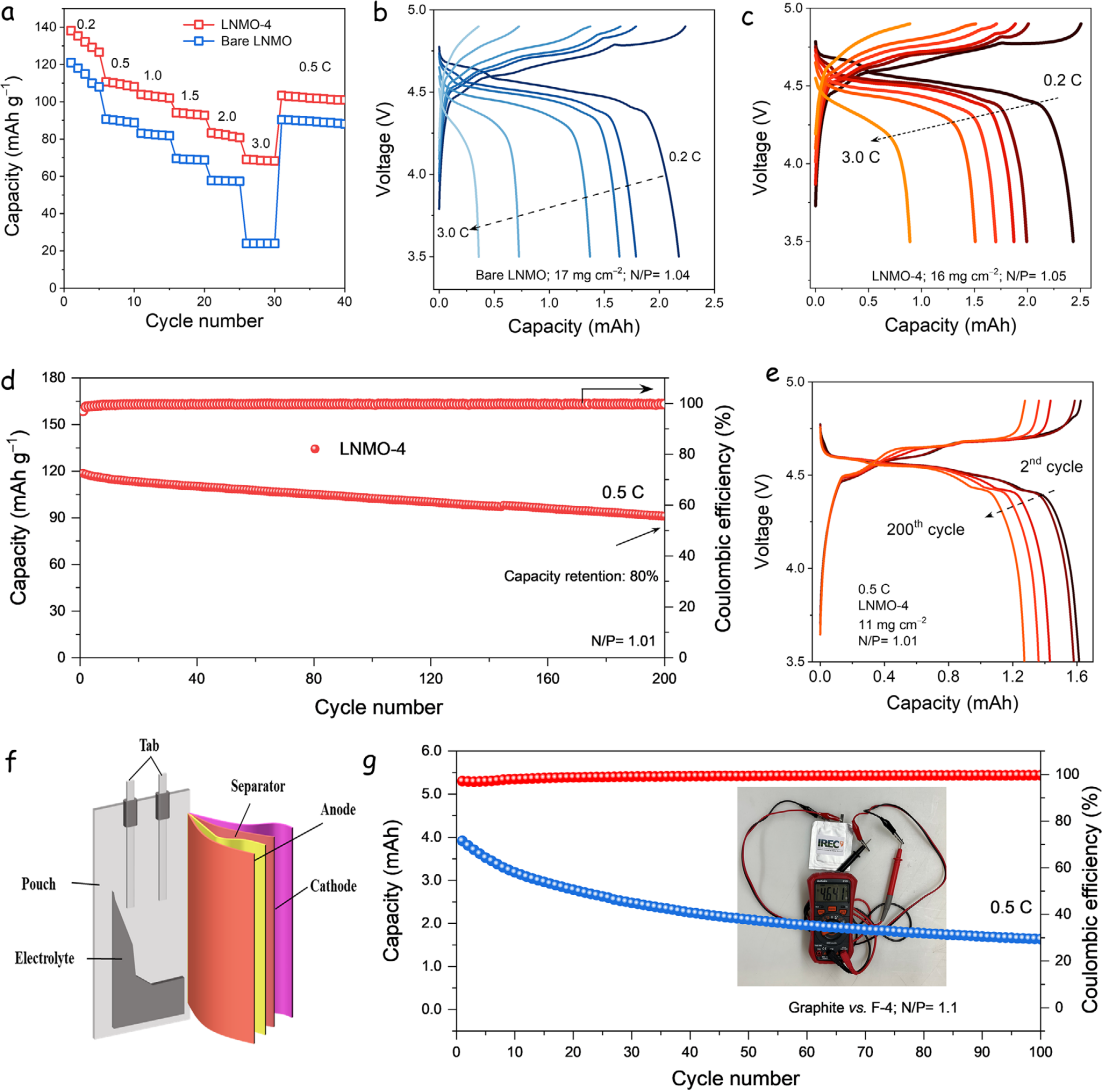
Electrochemical performance of graphite // LNMO full cells. a) Rate performance. b,c) GCD at the different current densities of graphite // bare LNMO (b) and graphite//LNMO‐4 (c) cells. d) Cycling performance of graphite // LNMO‐4 at 0.5C. e) corresponding GCD curves. f) Schematic diagram of the graphite//LNMO pouch cell with one‐piece anode and cathode. g) Cycling performance of the pouch cell at 0.5C, and the inset shows an image of the pouch cell and its output voltage after 100 cycles.

The electrochemical performance of the LNMO electrodes was further evaluated under high mass loading conditions using graphite as the anode with a diameter of 12 mm (specific capacity of 350 mAh∙g^−1^) to reflect their potential in practical full‐cell applications better. **Figure** **7**a compares the rate capabilities of the bare LNMO (17 mg∙cm^−2^, N/P ratio = 1.04) and LNMO‐4 (16 mg∙cm^−2^, N/P ratio = 1.05) cathodes. The LNMO‐4 electrode consistently outperforms the bare LNMO across all current rates, delivering reversible specific capacities of 104 mAh·g^−1^ at 1.0C and 82 mAh·g^−1^ at 2.0C, compared to 90 and 61 mAh·g^−1^ for the bare LNMO, respectively. The corresponding GCD profiles, shown in Figure 7b,c for bare LNMO and LNMO‐4, further support these findings. The LNMO‐4 electrode exhibits notably higher current densities and improved voltage profiles, particularly in the range of 0.2C–2.0C. This highlights the typical high‐rate capability of spinel LNMO and the enhanced kinetics enabled by the AlF_3_/LiF modification. Figure 7d shows the long‐term cycling performance of graphite // LNMO‐4 full cell at 0.5C, using a mass loading of 11mg·cm^‒2^ for the cathode and a loading of 4.5 mg·cm^‒2^ for the anode, corresponding to a N/P ratio of 1.01. The LNMO‐4 full cell demonstrates remarkable cycling stability, retaining 80% of its initial capacity after 200 cycles. Figure 7e presents the GCD profiles of the LNMO‐4 full‐cell at the 2nd, 50th, 100th, 150th, and 200th cycles, illustrating excellent voltage stability and capacity retention throughout prolonged cycling. The GCD curves exhibit the distinctive electrochemical features of disordered LNMO, characterized by a reversible spinel structure.^[^ ^20^ ^]^ These factors collectively contribute to the enhanced performance and long‐term stability of the LNMO‐4 electrode in full‐cell configurations.

To further assess the practical applicability of the LNMO‐4 material, a pouch cell was assembled using a cathode with a mass loading of 8.2 mg∙cm^‒2^. Figure 7f provides a schematic diagram of the pouch cell configuration, illustrating the integration of an LNMO‐4 cathode material with a graphite anode of 3.6 mg∙cm^‒2^. The pouch cell maintained a working voltage of 4.6 V after 100 cycles, as shown in the inset photograph in Figure 6g. The cell delivered an initial reversible capacity of 121 mAh·g^‒1^, demonstrating strong electrochemical performance under practical operating conditions and underscoring its potential for integration into high‐voltage LIB systems.

## 3 Conclusion

In summary, we presented a comprehensive study aimed at enhancing the electrochemical performance of disordered LNMO through a scalable and straightforward solid‐state synthesis approach. By adjusting synthesis amounts and preparing reference samples to understand the individual contributions of AlF_3_ and LiF, we fine‐tuned the physicochemical, structural, and electrochemical properties of the LNMO cathode. The synergy between AlF_3_ and LiF was designed to promote fully reversible lithiation/delithiation, mitigate irreversible phase transitions, and boost Li^+^ diffusion, thereby allowing LNMO to approach its theoretical capacity and exhibit excellent rate capability, while enhancing its structural stability for prolonged cycle life. The optimized LNMO‐4 (10 mg AlF_3_ + 30 mg LiF) sample exhibits outstanding performance, delivering high reversible capacities of 141.2, 139.1, 129.2, 120.6, 110.3, 93.5, and 76.1 mAh∙g^‒1^ at 0.2C, 0.5C, 1.0C, 2.0C, 3.0C, 4.0C, and 5.0C, respectively. In full‐cell configurations paired with graphite and utilizing high cathode mass loadings (11.0 mg∙cm^‒2^), LNMO‐4 maintains 80% capacity retention after 200 cycles at 0.5C, within a wide voltage window of 3.5–4.9 V. Notably, even under practical high‐loading conditions, the LNMO‐4 cathode demonstrates long‐term cycling stability, and pouch cell testing further validates its potential for commercial applications. Results are supported by additional sampling used to deconvolute individual contributions of AlF_3_ and LiF. The LNMO‐Al (10 mg AlF_3_) sample exhibits superior capacity retention across a wide range of C‐rates. In comparison, the LNMO‐Li (30 mg LiF) sample delivers enhanced electrochemical stability, maximizing cycle life. This highlights the synergy between AlF_3_ and LiF, resulting in an exceptional electrochemical performance for the optimized LNMO‐4 electrode. To understand the origin of this enhanced cycling performance, *operando* XAS and SXRD were conducted to monitor valence state evolution and structural changes during cycling, revealing that the bare LNMO experiences significant structural degradation associated with its reaction mechanism involving a large two‐phase domain (rock‐salt + spinel) in Li_1−_ *_x_* Ni_0.5_Mn_1.5_O_4_ during discharge, particularly involving the Ni^4+^/Ni^3+^ redox couple. In contrast, LNMO‐4 with optimized AlF_3_/LiF modifications exhibits a narrower two‐phase domain, smoother valence transitions, and preservation of the spinel structure, resulting in reduced electrochemical polarization, faster Li⁺ kinetics, and higher structural reversibility, thereby approaching the theoretical capacity with fast Li^+^ diffusion in disordered LNMO. Overall, this work demonstrates a practical and scalable design strategy for realizing high‐energy‐density LNMO cathodes that approach their theoretical capacity, enabled by suppressing the rock‐salt phase and stabilizing the disordered LNMO structure. The findings highlight the importance of using operando techniques to provide valuable insights into the reaction mechanism of intercalation materials, helping to design and optimize LNMO cathode materials with enhanced cycle life. These insights pave the way for the future development of high‐voltage spinel cathode materials in next‐generation LIBs.

## Conflict of Interest

The authors declare no conflict of interest.

## Author Contributions

X.C. designed the experiments, investigated, and performed the formal analysis. C.E., A.P.B., and E.M. supported the operando battery measurements. S.H. and M.I. conducted the TEM analysis, X.L. and J.L. supported the physicochemical characterizations, X.C., A.C., and J.J.B. co‐wrote the manuscript, J.L. and M.I. reviewed and edited the manuscript, J.J.B. administered the project, resources, and the funding.

## Supporting information



Supporting Information

## Data Availability

The data that support the findings of this study are available from the corresponding author upon reasonable request.
