# Supplementary material for: Mitigating the Rock‐Salt Phase Transformation in Disordered LNMO Through Synergetic Solid‐State AlF3/LiF Modifications
Source: Adv Sci (Weinh). 2025 Dec 12;13(11):e15962. doi: 10.1002/advs.202515962 (PMC12931164; doi:10.1002/advs.202515962)
Supplement: Supplementary file 1 — Supporting Information [file ADVS-13-e15962-s001.docx]

**SUPPORTING INFORMATION**

**Mitigating the Rock-Salt Phase Transformation in Disordered LNMO Through Synergetic Solid-state AlF_3_ /LiF Modifications**

*Xingqi Chang ^a, b^, Carlos Escudero ^c^, Ashley P Black ^c^, Sharona Horta ^d^, Elías Martínez ^a^, Xuan Lu ^a, e^, Jordi Llorca ^e^, Maria Ibañez ^d^, Jordi Jacas Biendicho ^a^ *, and Andreu Cabot ^a, f^ **

^a^ Catalonia Institute for Energy Research – IREC, Sant Adrià de Besòs, Barcelona 08930, Spain

^b^ Universitat de Barcelona, Carrer de Martí i Franquès, Barcelona 08028, Spain

^c^ ALBA Synchrotron Light Source, 08290, Cerdanyola del Vallès, Spain

^d^ IST Austria, Am Campus 1, 3400 Klosterneuburg, Austria

^e^ Department of Chemical Engineering, Universitat Politècnica de Catalunya, EEBE, Eduard Maristany 10-14, 08019 Barcelona, Spain

^f^ ICREA Pg. Lluis Companys, 08010 Barcelona, Catalonia, Spain

Email: J. J. Biendicho: jjacas@irec.cat; A. Cabot: acabot@irec.cat;

**Experimental section:**

*Chemicals:* Whatman glass fiber (GF/A), N-methyl-2-pyrrolidone (NMP), aluminum fluoride (AlF_3_, 99%), and lithium fluoride (LiF, 99%) were purchased from Sigma-Aldrich. Mili-Q water (18 MΩ cm^-1^) was supplied by a Purelab Flex from Elga. Ethanol was of analytical grade and obtained from Lestlab Delivering Solutions L.U. Carbon black, graphite anode powder, Celgard 2400 separator, sodium carboxymethyl cellulose (CMC), and polyvinylidene fluoride (PVDF) were purchased from Guangdong Canrd Co., Ltd. Graphite anode powder and 1.0 M LiPF_6_ in EC-DEC (1:1, v/v) were purchased from Sigma-Aldrich. All the chemicals were used as received without further purification. TOPSOE Company (Denmark) provided the disordered phase LiNi_0.5_Mn_1.5_O_4_ (LNMO) powder

*Synthesis of LNMO:* A certain proportion of LNMO, AlF_3_, and LiF was placed in an onyx ball milling jar (more detailed information was shown in Table S1) and ball milled in a planetary ball mill at 300 rpm for 4 h. Subsequently, it was annealed in an air atmosphere at 800 °C for 4 h.

*Materials characterization:* The samples were characterized through transmission electron microscopy (TEM) and high-resolution TEM (HRTEM) using a JEOL JEM-3200FS, operating at 200 kV with a point-to-point resolution of 0.19 nm. A JEOL JEM-3200FS field emission TEM equipped with an Oxford Instruments EDS analyzer was used for the EDS of TEM. X-ray photoelectron spectroscopy (XPS) on a SPECS system with an Al anode XR50 source operated at 150 W and a Phoibos 150 MCD-9 detector was used to analyze the surface elemental composition and chemical states. The pressure in the analysis chamber was consistently below 10^-7^ Pa. The area analyzed was 2$\times$2 mm. The pass energy of the hemispherical analyzer was set at 25 eV, and the energy step was 0.1 eV. Data processing was performed with the Multipak data reduction software (Physical Electronics-PHI, Inc., EE.UU.). Binding energy values were corrected using the C 1s peak at 284.8 eV.

Both XAS and SXRD sequential measurements were acquired at BL-16-NOTOS beamline (BL-16) at ALBA Synchrotron Light Source (ALBA) in Cerdanyola del Vallès (Barcelona, Spain). The X-ray absorption spectroscopy (XAS) and synchrotron X-ray diffraction (SXRD) data were recorded at room temperature using CR2032-type coin cells with 75 µm Kapton windows, allowing the synchrotron beam to pass through. The XAS measurements around the Mn and Ni K-edges (6539 and 8333 eV, respectively) were acquired in transmission mode using ionization chambers from Oken. The SXRD measurements were acquired with a wavelength of λ = 0.954 Å (13 keV) in transmission mode (Debye-Scherrer geometry) with a 6 module Mythen-I detector from PSI-DECTRIS with a 2θ range from 5° to 55°, was refined with GSAX-II .

*Electrochemical measurements:* Electrochemical tests of the batteries were performed on CR2032-type coin cells. The LNMO cathode electrode was prepared by spreading a slurry of 90 wt.% active material, 5 wt.% carbon black, and 5 wt.% PVDF binder in NMP on an Aluminum foil, drying at 110 °C overnight in a vacuum oven. Subsequently, the cathode electrode was cut to a diameter of 12 mm. The loading mass of active materials was kept constant at 4.0 to 5.0 mg‧cm^–2^ for half cells. For *operando* measurements at BL-16-NOTOS beamline, the cathode loadings of LNMO and LNMO-4 were ~8 mg‧cm^–2^. Coin cells were fabricated using a lithium disc with a diameter of 15.6 mm as an anode, LNMO electrode with a diameter of 12 mm as the cathode, Celgard-2400 with a diameter of 18 mm as a separator, and 1.0 M LiPF_6_ in EC-DEC (1:1, v/v) as the liquid electrolyte.

The full cells were assembled with a graphite anode (Φ = 12 mm), prepared by spreading a slurry of 90 wt.% graphite powder, 5 wt.% carbon black, and 5 wt.% CMC binder in H_2_O on a Copper foil, drying at 110 °C overnight in a vacuum oven. The areas of the anode and cathode of the pouch cell are 4.0 cm^2^, coupled with the anode loading of 3.6 mg∙cm^‒2^ and cathode loading of 8.2 mg∙cm^‒2^.

All coin and pouch cells were assembled in the glovebox and measured at room temperature. Before cycling, all cells were rested for 8 hours to ensure sufficient electrolyte penetration. GCD tests were conducted on the Neware BES-4008 battery test system, with a potential range set between 3.5 V and 4.9 V (vs. Li^+^/Li). Cyclic voltammetry (CV) was performed on a BCS-810 battery tester from Bio-Logic at different scan rates with a potential range set from 3.5 V to 4.9 V (vs. Li^+^/Li). Electrochemical impedance spectroscopy (EIS) measurements were carried out using a sinusoidal voltage with an amplitude of 10 mV and a frequency from 10 kHz to 0.10 Hz. Operando electrochemical data sets collected at BL-16-NOTOS beamline were measured using a Bio-Logic tester in the voltage window of 3.5 to 4.9 V at 0.1 C.

**Table S1**. Materials ratio for the sample synthesis.

| **sample** | **LNMO-1** | **LNMO-2** | **LNMO-3** | **LNMO-4** | **LNMO-5** | **LNMO-Air** | **LNMO-Al** | **LNMO-Li** |
| --- | --- | --- | --- | --- | --- | --- | --- | --- |
| **LNMO** | 2.0 g | 2.0 g | 2.0 g | 2.0 g | 2.0 g | 2.0g | 2.0 g | 2.0 g |
| **AlF_3_** | 40 mg | 30 mg | 20 mg | 10 mg | 0 | 0 | 10 mg | 0 |
| **LiF** | 0 | 10 mg | 20 mg | 30 mg | 40 mg | 0 | 0 | 30 mg |

**
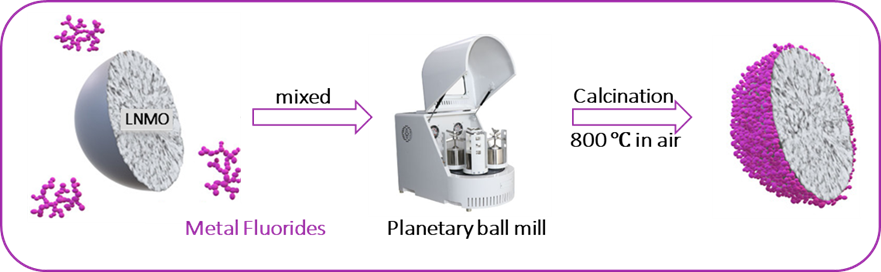
**

,

**Figure S1**. Scheme of the synthesis procedure of LNMO samples.


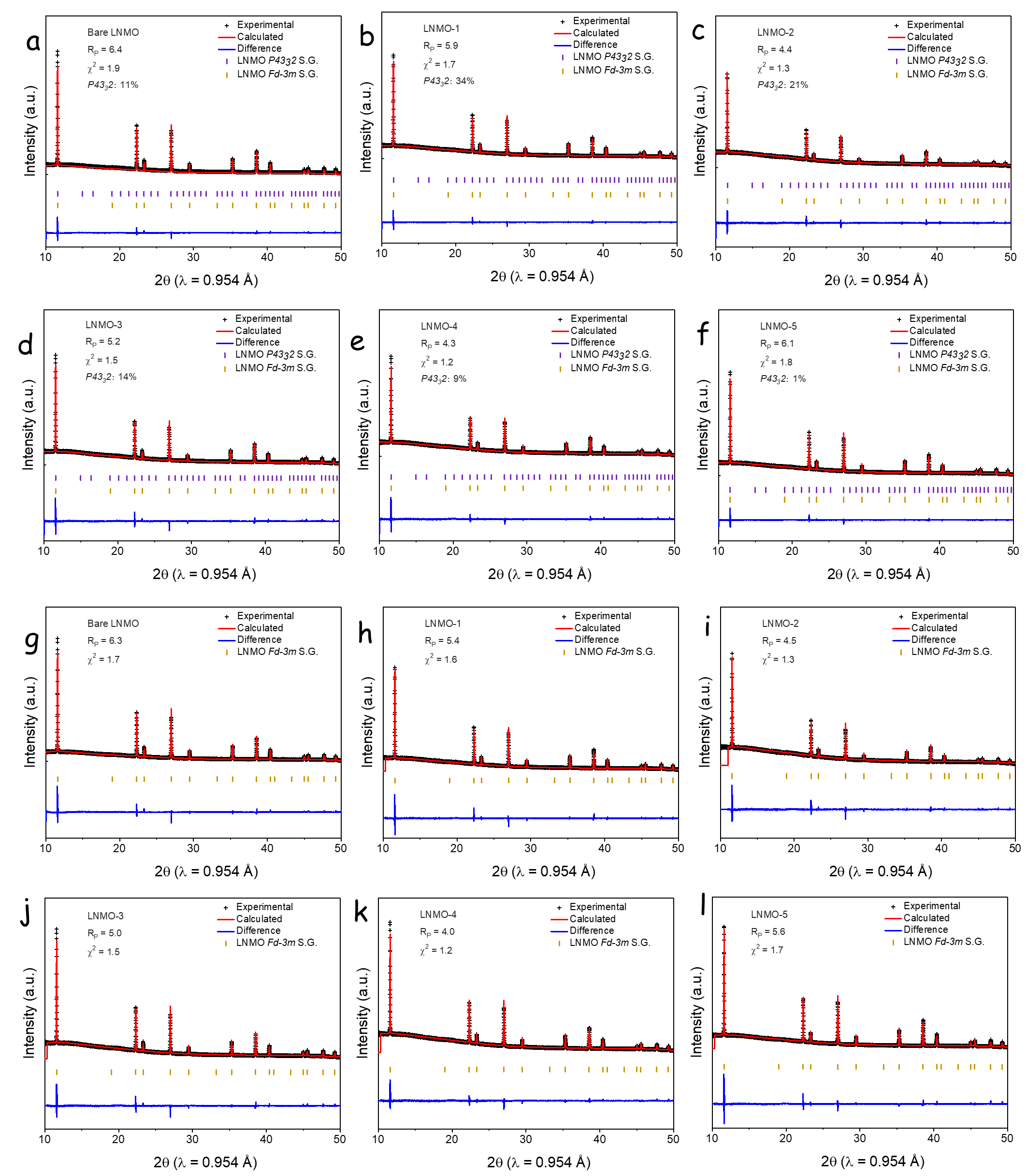


**Figure S2.** Rietveld refinement analysis of the SXRD pattern for LNMO samples. (a-f) SXRD Rietveld refinement data based on the *F3-dm* and *P4332* phase: (a) bare LNMO, (b) LNMO-1, (c) LNMO-2, (d) LNMO-3, (e) LNMO-4, and (f) LNMO-5. (g-l) SXRD Rietveld refinement data based on the *F3-dm* phase: (g) bare LNMO, (h) LNMO-1, (i) LNMO-2, (j) LNMO-3, (k) LNMO-4, and (l) LNMO-5.

**Table S2**. Structural and well-of-fit model parameters for LNMOs obtained from Rietveld refinement of SXRD data

| Sample | Two-phase refinement | | | | | *F3-dm* phase refinement | | | |
| --- | --- | --- | --- | --- | --- | --- | --- | --- | --- |
|  | R_P_ | χ^2^ | Lattice  (*F3-dm*) | Lattice  (*P43_3_2*) | *P43_3_2* (wt.%) | R_P_ | χ^2^ | Lattice  (*F3-dm*) |  |
| Bare LNMO | 6.4 | 1.9 | 8.1797 Å | 8.1645 Å | 11 | 6.3 | 1.7 | 8.1725 Å |  |
| LNMO-1 | 5.9 | 1.7 | 8.1785 Å | 8.1685 Å | 34 | 5.4 | 1.6 | 8.1782 Å |  |
| LNMO-2 | 4.4 | 1.3 | 8.1740 Å | 8.1614 Å | 21 | 4.4 | 1.1 | 8.1779 Å |  |
| LNMO-3 | 5.2 | 1.5 | 8.1793 Å | 8.1740 Å | 14 | 5.0 | 1.4 | 8.1736 Å |  |
| LNMO-4 | 4.3 | 1.2 | 8.1695 Å | 8.1674 Å | 9 | 4.0 | 1.2 | 8.1697 Å |  |
| LNMO-5 | 6.1 | 1.8 | 8.1733 Å | 8.1645 Å | 1 | 5.6 | 1.7 | 8.1657 Å |  |


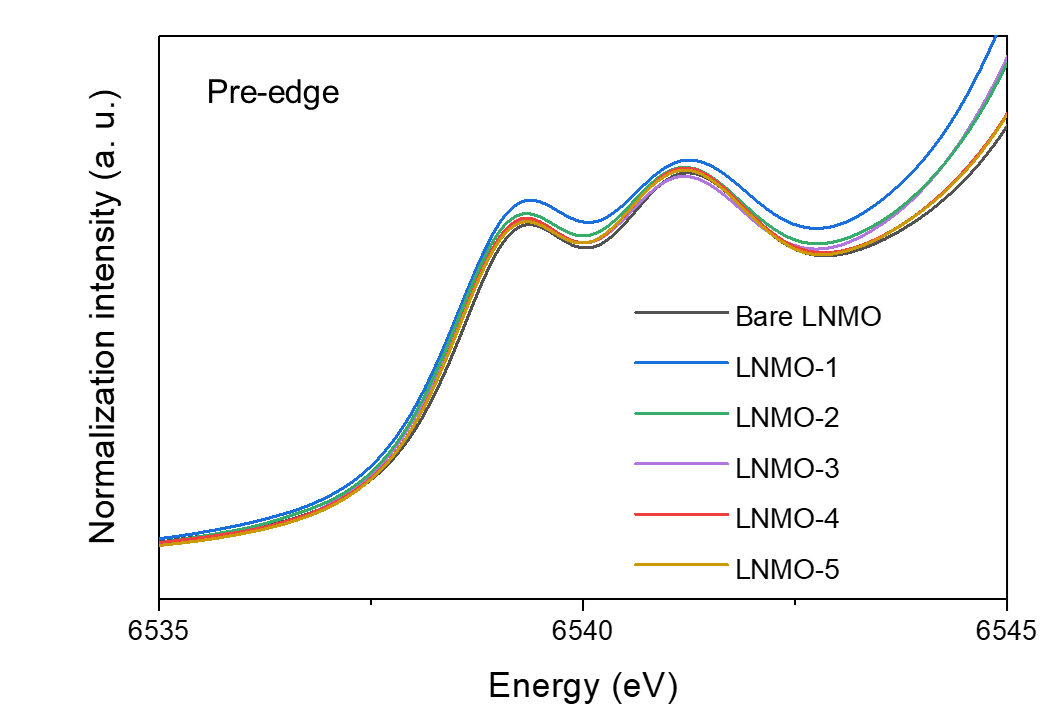


**Figure S3.** The Mn pre-edge XANES spectra of LNMO samples, attributed to the 1*s* to 3*d*.


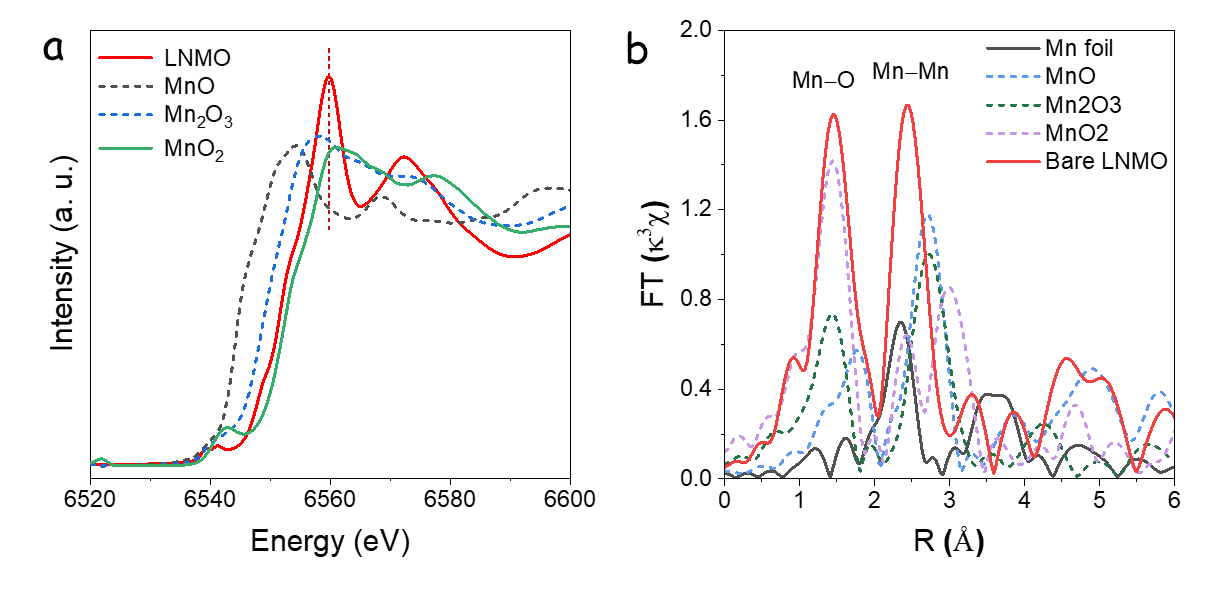


**Figure S4.** Mn K-edge XAS data of bare LNMO with the standard materials (i.g, Mn foil, MnO, Mn_2_O_3_, and MnO_2_). (a) XANES spectra. (b) FT-EXAFS fitting in R space, the peaks at ~1.4 and ~2.4 Å correspond to the Mn−O and Mn−Mn bonds, respectively.


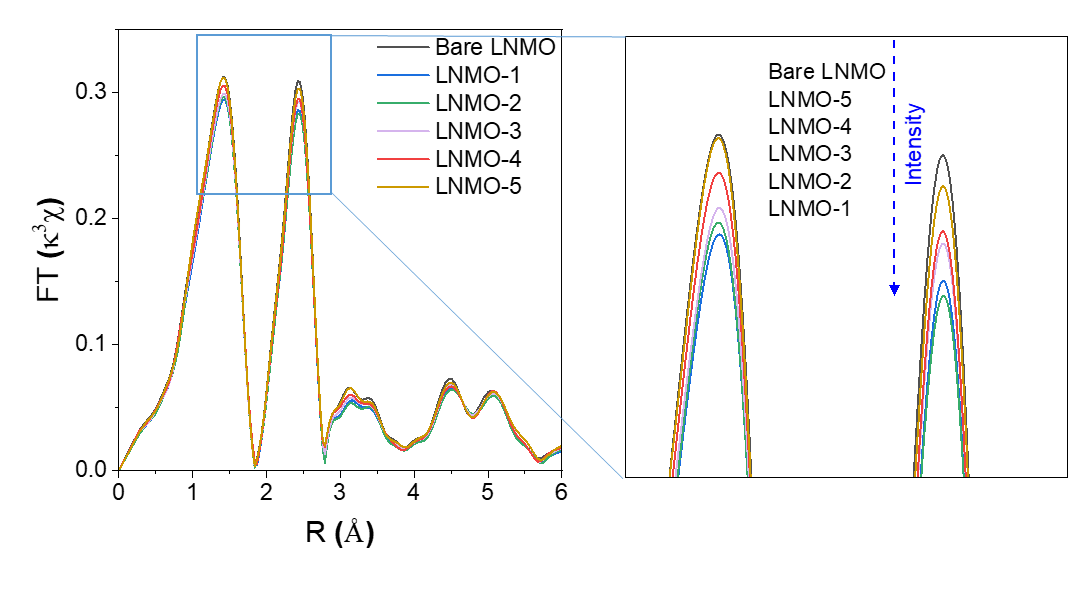


**Figure S5**. Fourier-Transformed (FT) EXAFS spectra for the Mn K-edge. An enlarged region marked with a blue box is shown on the right side; the intensity of the first peak decreases as: bare LNMO > LNMO-5 > LNMO-4 > LNMO-3 > LNMO-2 > LNMO-1.


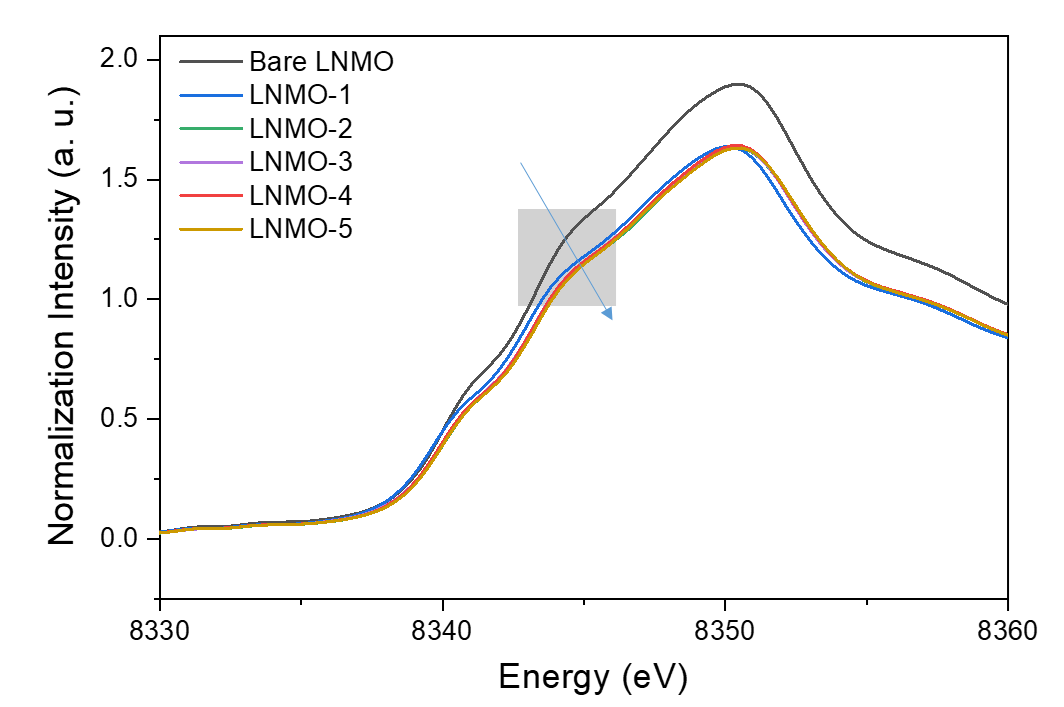


**Figure S6.** Pre-edge part of the Ni K-edge XANES of LNMO-Xs and bare LNMO.


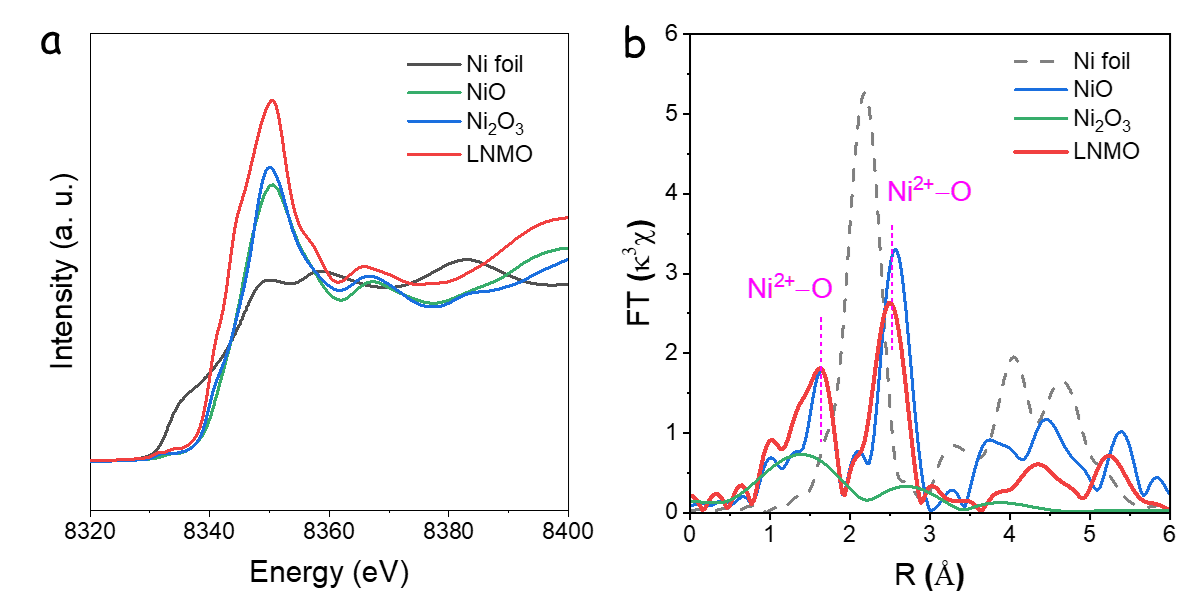


**Figure S7.** Ni K-edge XAS data of bare LNMO with the standard materials (i.g., Ni foil, NiO, and Ni_2_O_3_). (a) XANES spectra. (b) FT-EXAFS fitting in R space, the NiO material exhibits the two main peaks at ~1.6 and ~2.5 Å, consistent with the bare LNMO.


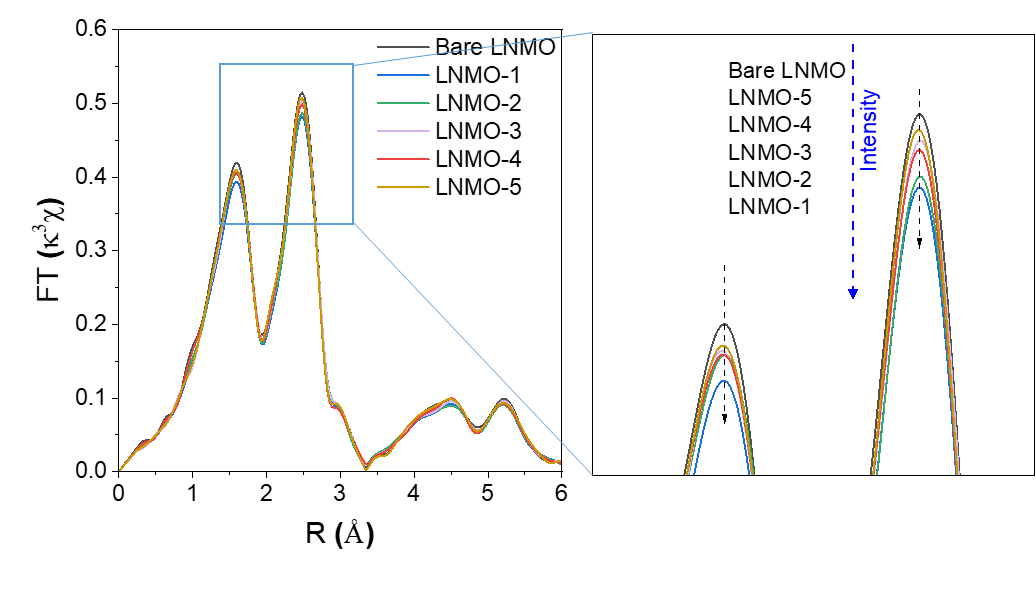
**Figure S8**. (a) Fourier-Transformed (FT) EXAFS spectra for the Ni K-edge. An enlarged region marked with a blue box is shown on the right side; the intensity of the first peak decreases as: bare LNMO > LNMO-5 > LNMO-3 > LNMO-4 > LNMO-2 > LNMO-1.


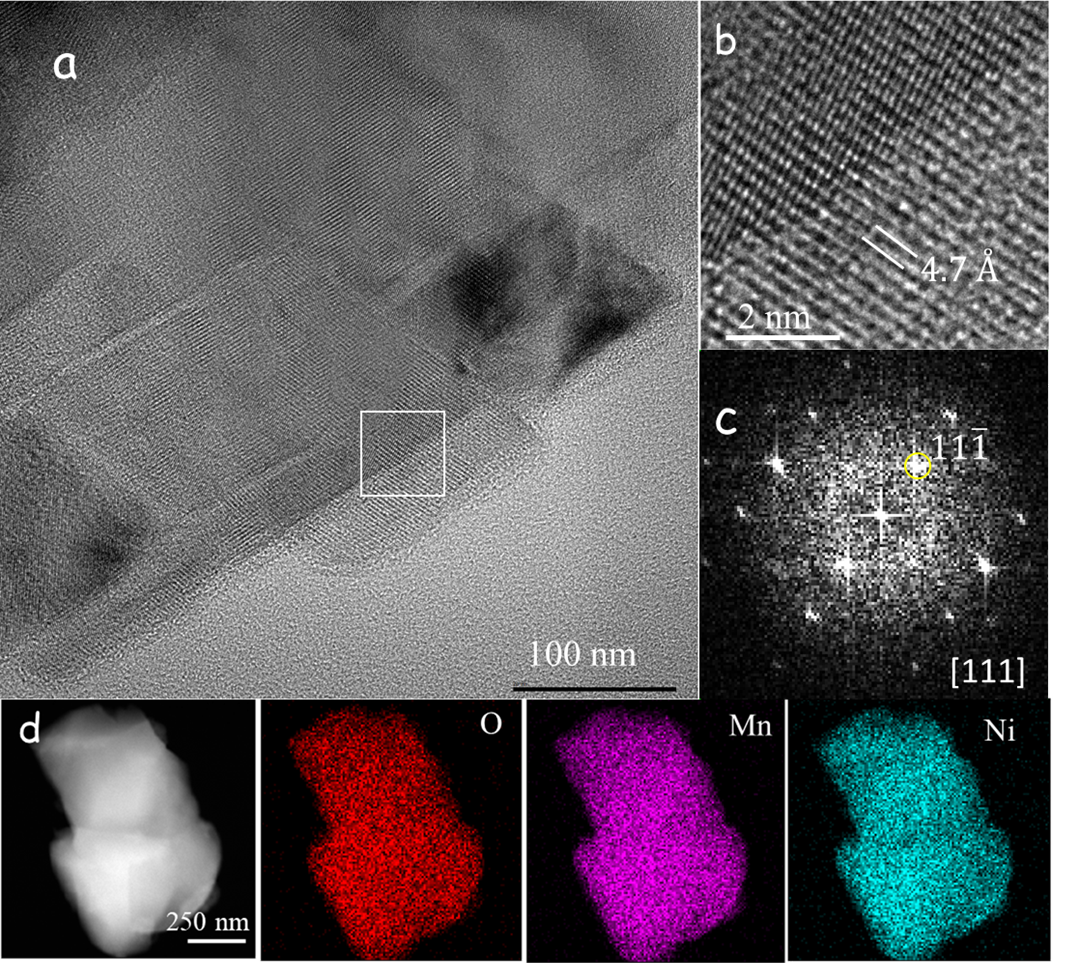


**Figure S9**. TEM images of bare LNMO. (a) TEM images. (b) HRTEM images and (c) the corresponding FFT images (d) EDS mappings.


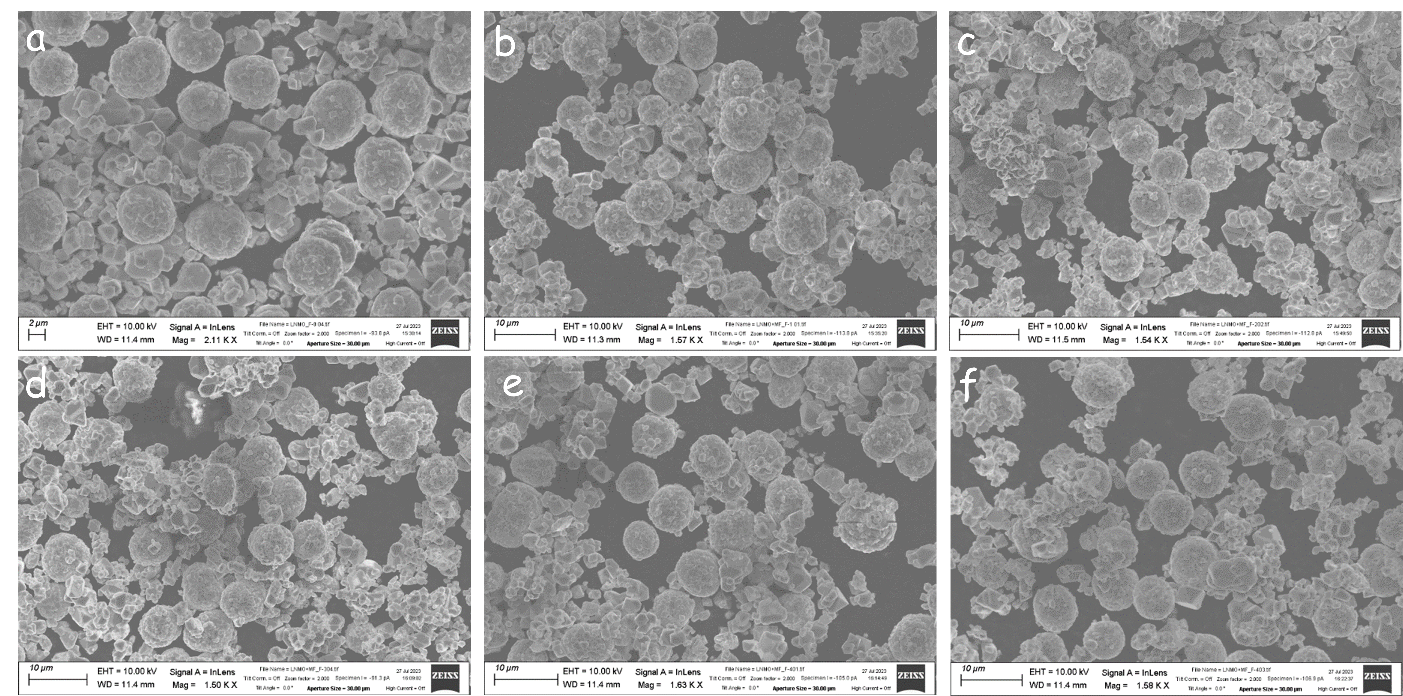


**Figure S10**. SEM images of samples. (a) Bare LNMO. (b) LNMO-1. (c) LNMO-2. (b) LNMO-3. (e) LNMO-4. (f) LNMO-5.

**Figure S11**. Equivalent circuit model.


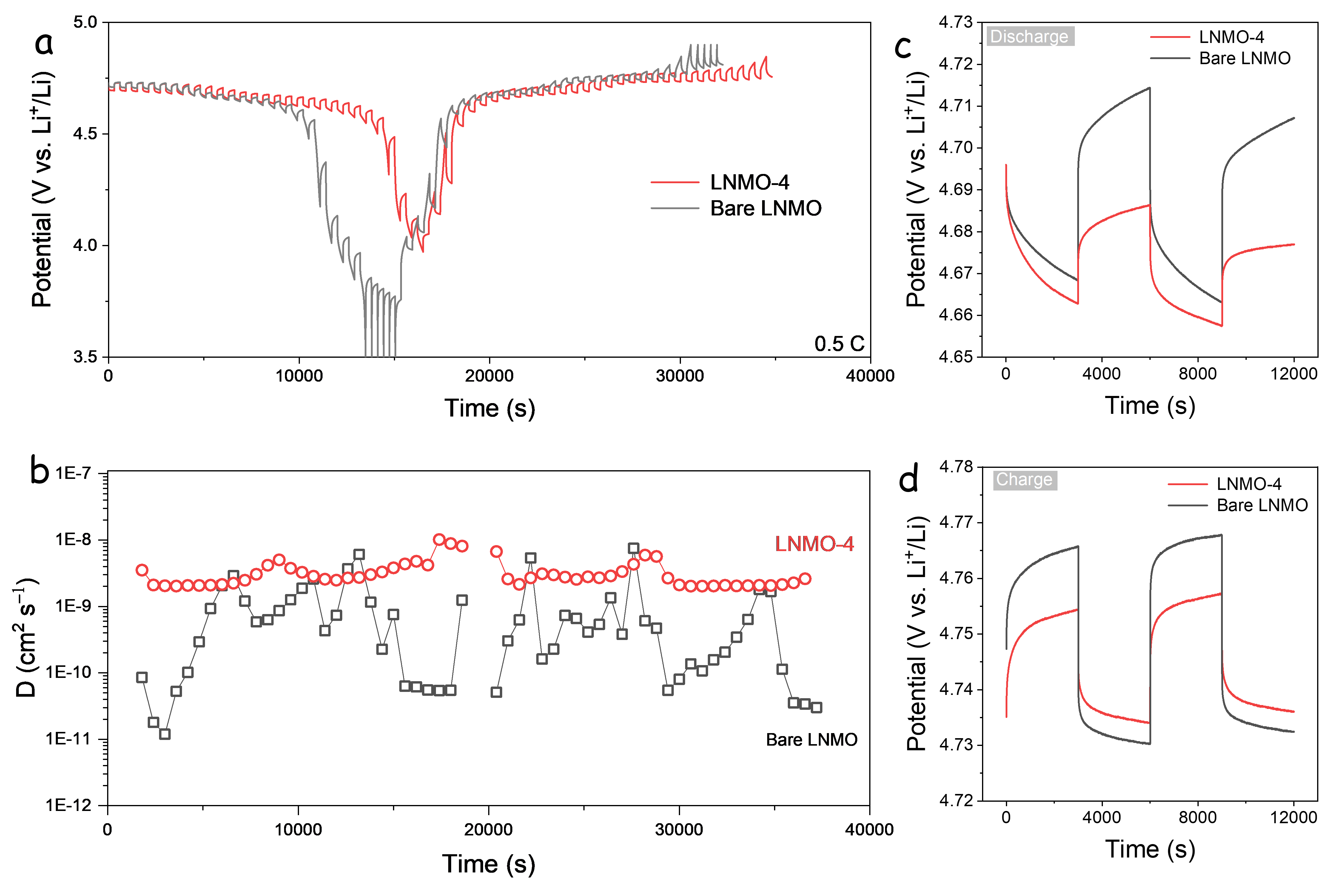


**Figure S12**. GITT results for bare LNMO and LNMO-4 with a 5 min relaxation time and galvanostatic process of 5 min carried out at current density of 0.5C. (a) Charge/discharge/rest profiles. (b) Li-ion diffusion coefficient (c) Discharge/rest profiles for discharge plateau located at 4.68 V. (d) Charge/rest profiles for charge plateau assigned to 4.75 V.


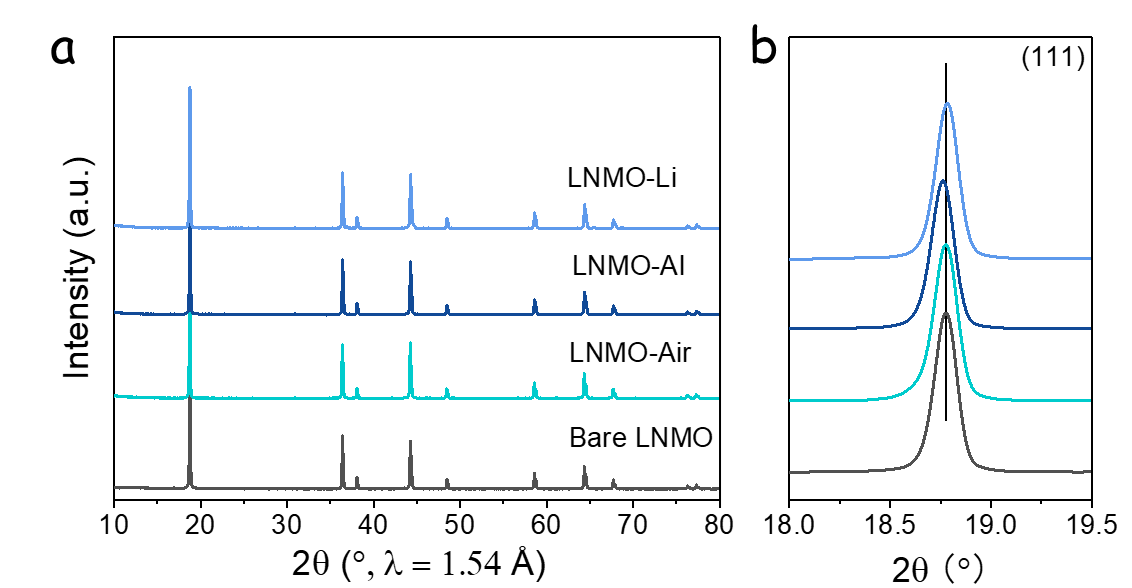


**Figure S13.** XRD patterns of (a) Bare LNMO, LNMO-Air, LNMO-Al, and LNMO-Li; (b) (111) peak of samples.


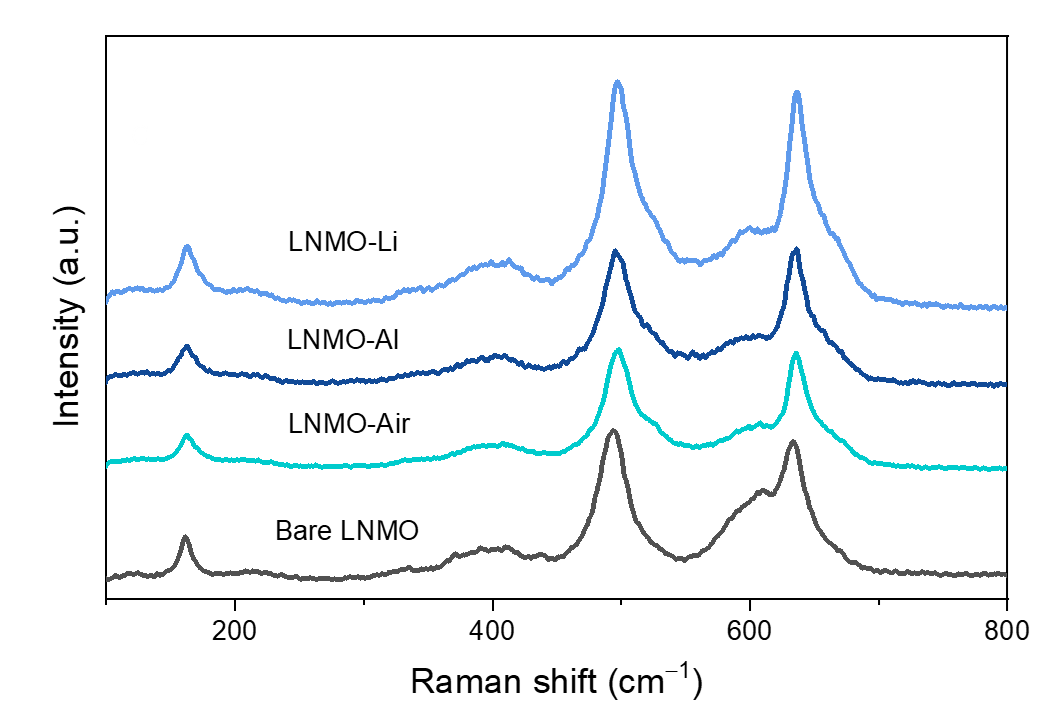


**Figure S14.** Raman spectra of bare LNMO, LNMO-Air, LNMO-Li, and LNMO-Al.


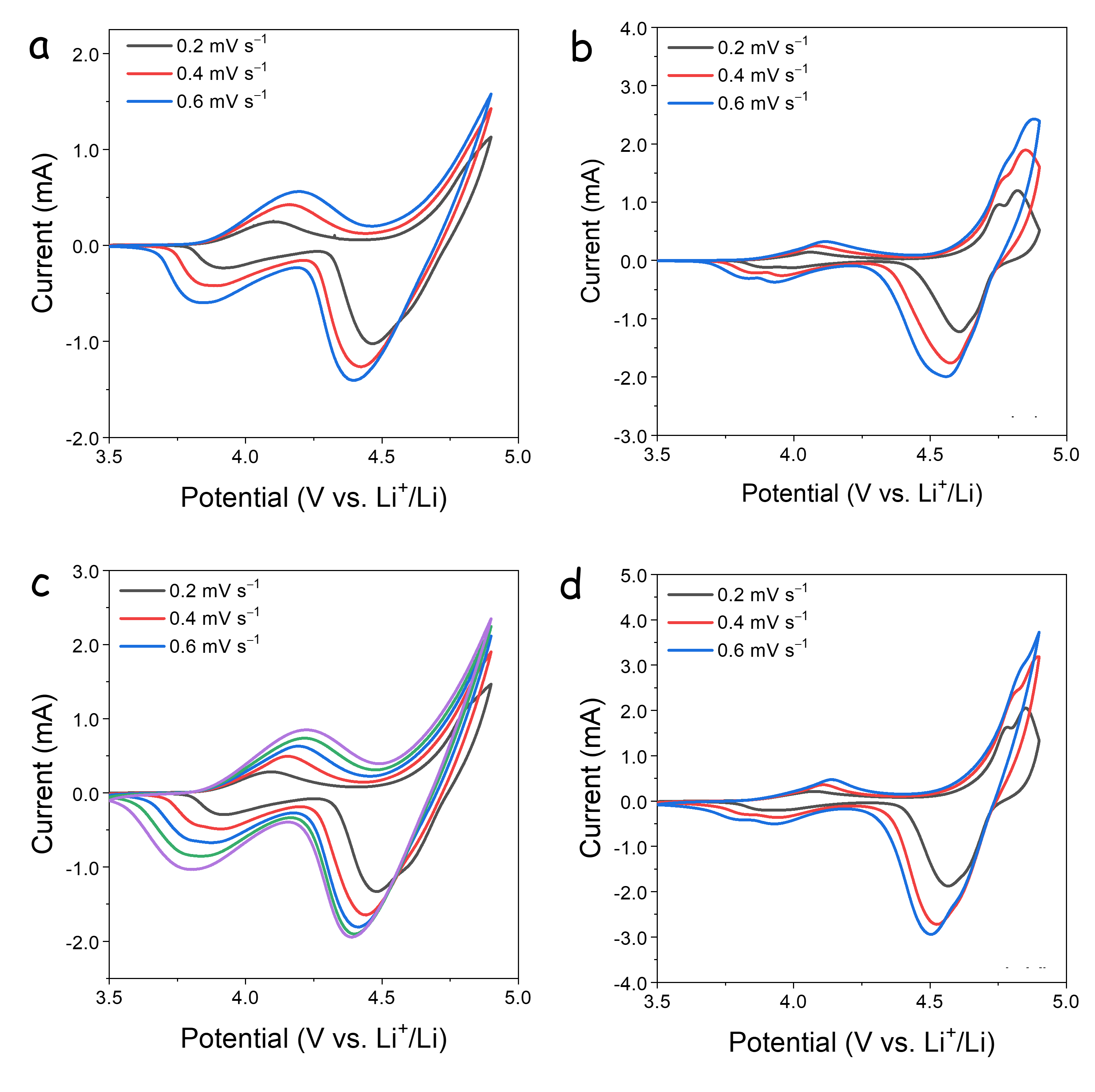


**Figure S15.** CV curves at the scan rates of 0.2, 0.4, and 0.6 mV·s^‒1^. (a) LNMO-Air. (b) LNMO-4. (c) LNMO-Li. (d) LNMO-Al.


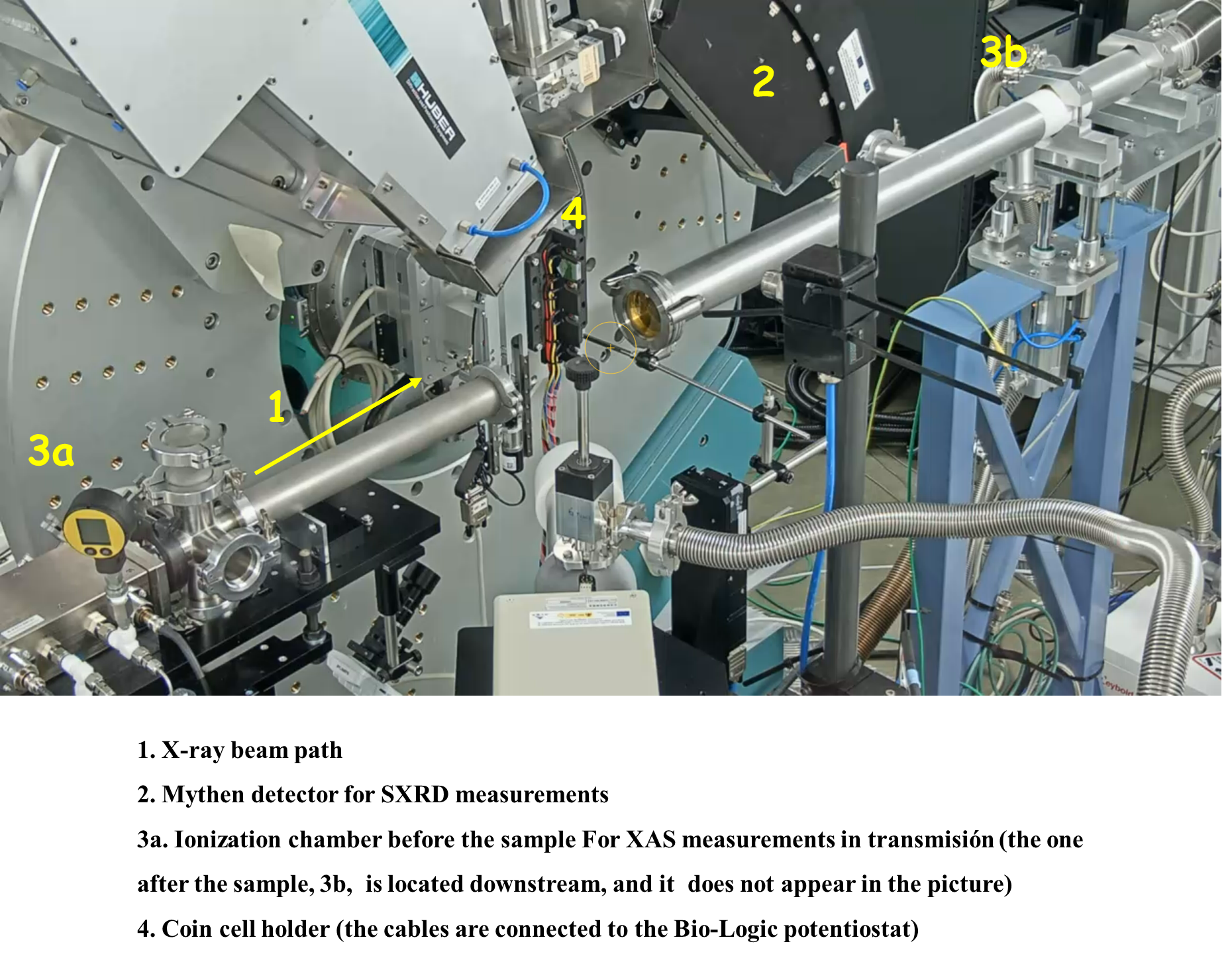


**Figure S16.** Picture of experimental setup used in BL16-NOTOS for the XAS and SXRD measurements, featuring *quasi-*simultaneous *operando* testing achieved through the alternating XAS and SXRD measurements.


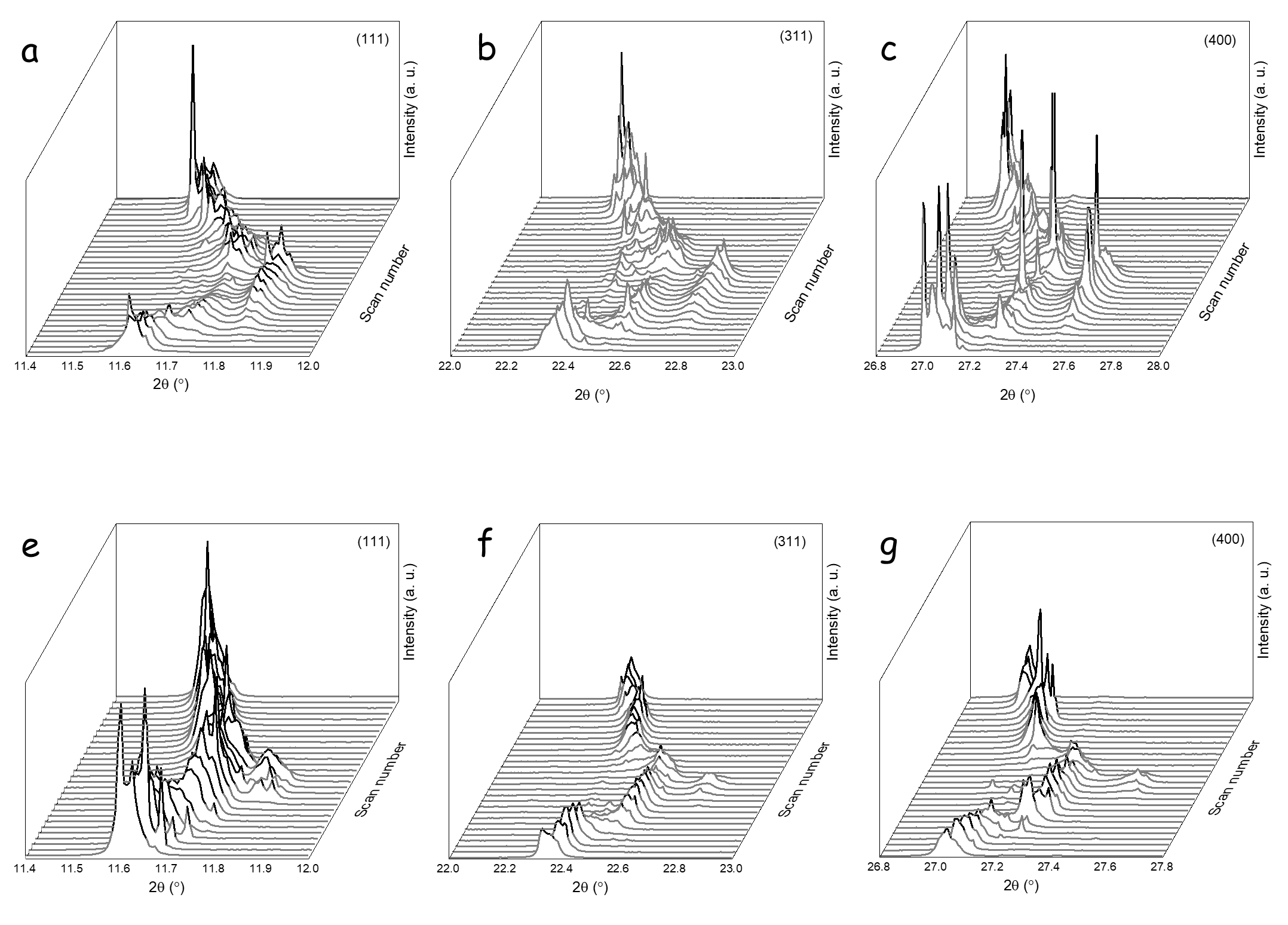


**Figure S17.** 3D waterfall diagram of *operando* SXRD tests under the galvanostatic charge/discharge test in the initial cycle carried out with a current of 0.1 C. (a‒c) correspond to the (111), (311), and (400) peaks for bare LNMO electrodes, respectively. The LNMO-4 electrodes were tested at the (111), (311), and (400) peaks in (a‒c), respectively.


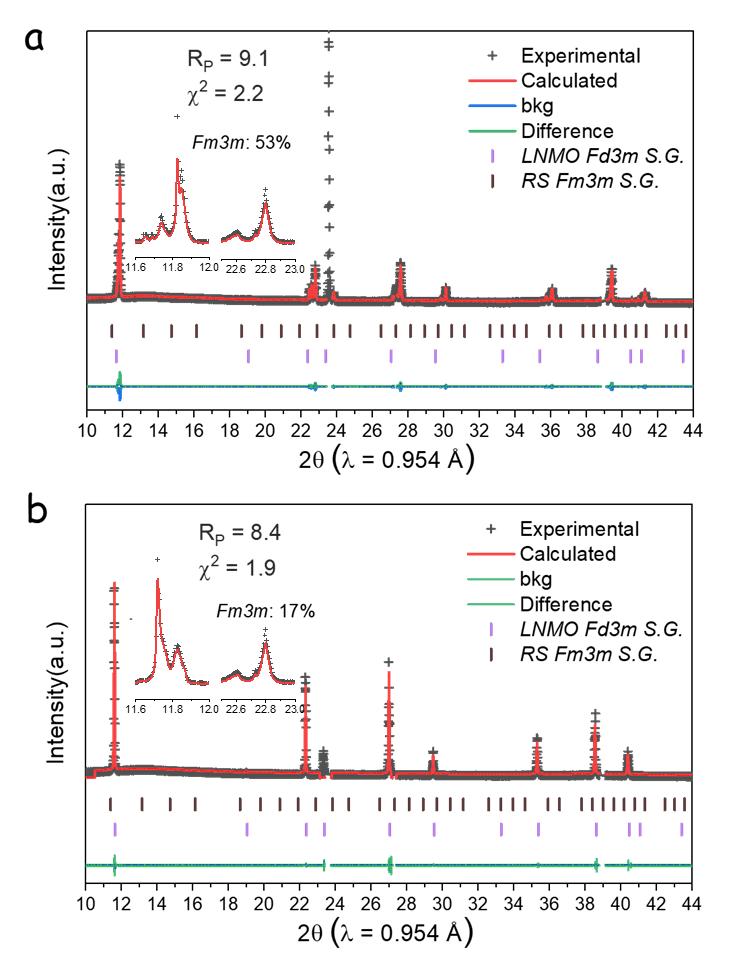


**Figure S18.** Rietveld refinement analysis of LNMO samples at the fully charged state; (a) Bare LNMO. (b) LNMO-4. Inset, a figure showing the (111) and (311) spinel peaks together with diffraction peaks for the rock-salt phase. The Al diffraction peaks from the coin cell were not considered in the analysis.


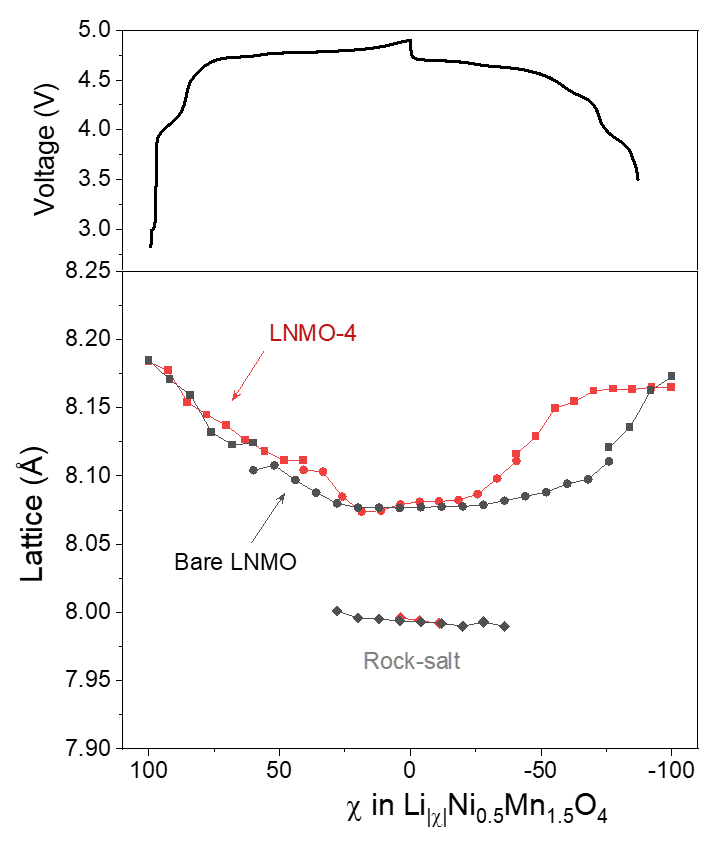


**Figure S19.** The lattice parameters of bare LNMO and LNMO-4 electrode during the first cycle, obtained from the operando SXRD data.


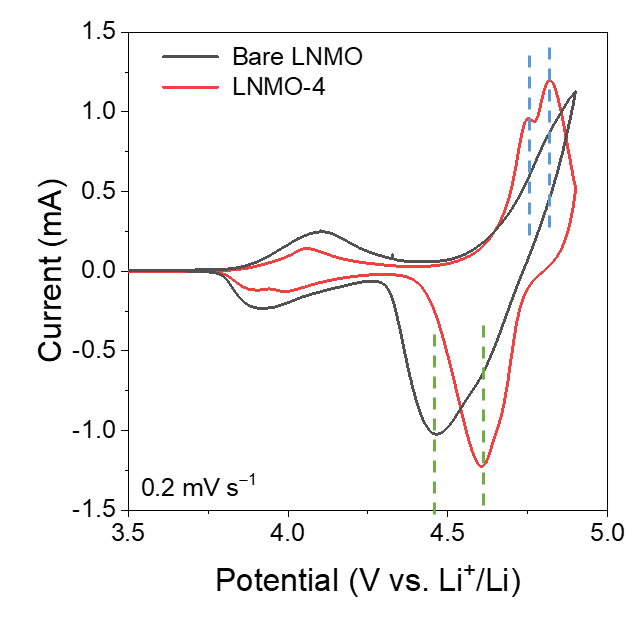


**Figure S20**. CV profiles for bare LNMO and LNMO-4 electrodes at the scan rate of 0.2 mV·s^‒1^.


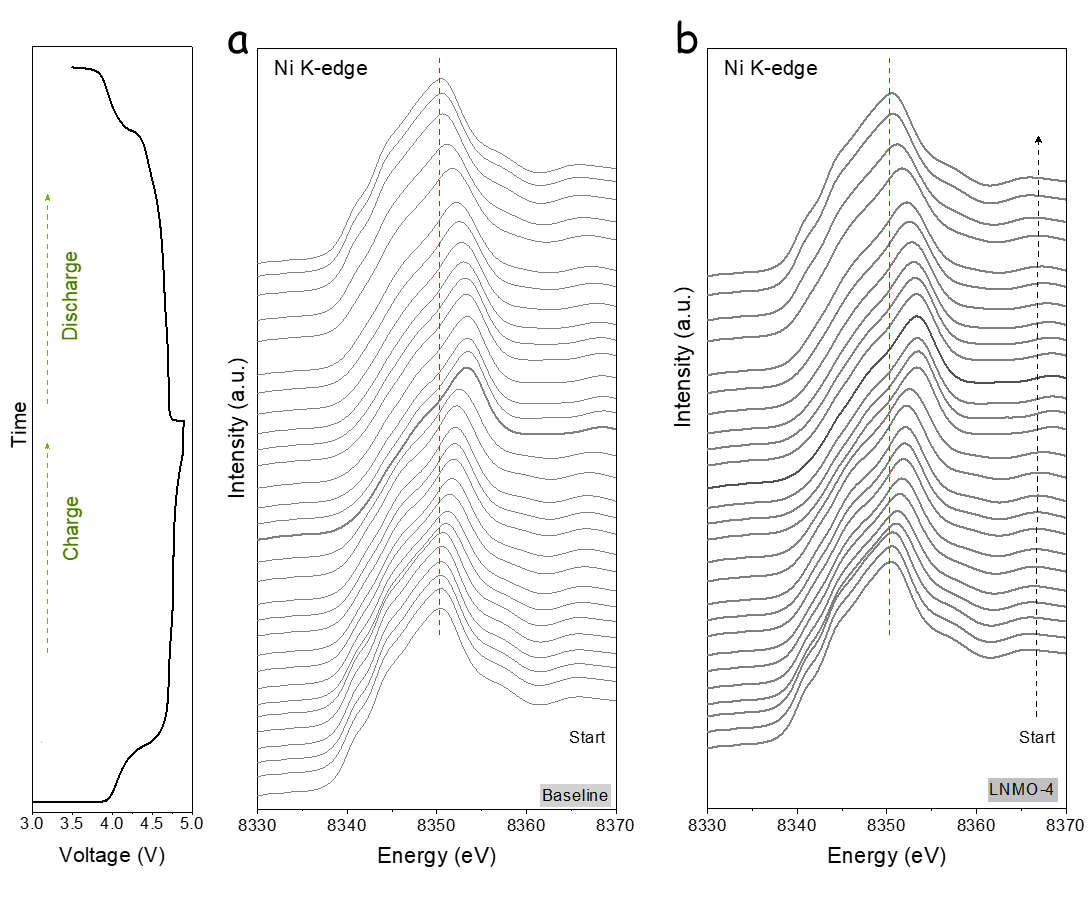


**Figure S21.** *Operando* Ni K-edge XAS spectra. (a) Bare LNMO electrode and (b) LNMO-4 sample under the GCD tests carried out with a current of 0.1C.
